# Human umbilical cord mesenchymal stem cell-derived treatment of severe pulmonary arterial hypertension

**DOI:** 10.1038/s44161-022-00083-z

**Published:** 2022-06-09

**Authors:** Georg Hansmann, Philippe Chouvarine, Franziska Diekmann, Martin Giera, Markus Ralser, Michael Mülleder, Constantin von Kaisenberg, Harald Bertram, Ekaterina Legchenko, Ralf Hass

**Affiliations:** 1 Department of Pediatric Cardiology and Critical Care, Hannover Medical School, Hannover, Germany; 2 European Pediatric Pulmonary Vascular Disease Network, Berlin, Germany; 3 Center for Proteomics and Metabolomics, Leiden University Medical Center (LUMC), Leiden, Netherlands; 4 Department of Biochemistry, Charité Universitätsmedizin Berlin, Berlin, Germany; 5 Departments of Obstetrics, Gynecology and Reproductive Medicine, Hannover Medical School, Hannover, Germany; 6 Biochemistry and Tumor Biology Lab, Department of Gynecology, Hannover Medical School, Hannover, Germany

**Keywords:** Vascular diseases, Mesenchymal stem cells

## Abstract

Here we report application of human umbilical cord mesenchymal stem cell (HUCMSC)-derived therapy for pulmonary arterial hypertension (PAH). A 3-year-old female presented with heritable PAH associated with hereditary hemorrhagic telangiectasia and was treated for 6 months with serial intravascular infusions of conditioned media (CM) from allogenic HUCMSCs. The treatment markedly improved clinical and hemodynamic parameters and decreased blood plasma markers of vascular fibrosis, injury and inflammation. A comparative analysis of single-cell RNA sequencing data collected from three HUCMSCs and two human umbilical vein endothelial cell (HUVEC) controls identified eight common cell clusters, all of which indicated regenerative potential specific for HUCMSCs. The properties of HUCMSCs were validated by untargeted label-free quantitation of the cell and CM proteome, suggesting increased activity of regeneration, autophagy and anti-inflammation pathways and mitochondrial function. Prostaglandin analysis demonstrated increased HUCMSC secretion of prostaglandin E2, known for its regenerative capacity. Additional prospective clinical studies are warranted to confirm and further explore the benefits of HUCMSC-derived therapy for PAH.

## Abstract (web summary)

Hansmann et al. show that serial intravascular infusions of conditioned media produced by allogenic umbilical cord mesenchymal stem cells markedly improved the clinical features of a young child with severe pulmonary arterial hypertension. The multi-omic approach analyzing the transcriptome and the proteome of these stem cells isolated from four donors provides initial insight into their beneficial paracrine function.

## Main

PAH is characterized by progressive, obliterative remodeling of pulmonary arterioles, pre-capillary vessel loss, right heart failure and death. The pathobiology of pulmonary vascular disease (PVD) and PAH is complex, multifactorial and driven by inflammation and metabolic dysfunction^1^. Despite remarkable improvements in pharmacotherapy^2,3^, advanced PAH is still a non-curable, debilitating and fatal condition^4^. Stem and progenitor cells and/or their secreted products may represent more efficient therapies for PAH^5,6^. Transforming growth factor beta (TGFβ) receptor superfamily members (bone morphogenetic protein receptor 2, *BMPR2*; activin A receptor-like type 1, *ACVRL1*; and endoglin, *ENG*) and their ligands play a critical role in the etiology of PAH^1,7–9^. Heterozygous, loss-of-function mutations in the *BMPR2*, *ACVRL1* and *ENG* genes, among others, have been described in familial/heritable PAH (HPAH) and idiopathic PAH (IPAH)^9^; such mutations are also found in hereditary hemorrhagic telangiectasia (HHT; Osler–Weber–Rendu disease). Patients with *ACVRL1* mutations who do develop PAH^7^ are particularly young, have often rapid disease progression and have a worse prognosis than patients with *BMPR2* mutations^10^. Here we demonstrate safe and efficient HUCMSC-derived treatment of severe, progressive PAH by means of serial intravascular infusions of HUCMSC-CM in one young patient with heritable PAH and HHT type 2 caused by an *ACVLR1* missense mutation.

## Results

At diagnosis, the 3-year-old girl was in critical condition, after two syncopal, ‘afebrile seizure episodes’, in World Health Organization (WHO) functional class 4, with a 6-minute walking distance (6MWD) of only 270 meters (SpO_2_ > 95%) and moderate thrombocytopenia at 8 × 10^3^ µl. She had a 10-month history of fatigue, repetitive nosebleeds (epistaxis) and mucocutaneous telangiectases at the lips, chest and lower extremities. Serum NTproBNP was greatly elevated at 2,414 ng l^−1^. Echocardiography showed severely compromised right ventricular systolic function (tricuspid annular plane systolic excursion (TAPSE), 1.4 cm) and tricuspid regurgitation, grade 2. The first diagnostic cardiac catheterization (CATH #0; treatment naive) was conducted in January 2019 at an external tertiary center. Invasive hemodynamic measurements demonstrated severe suprasystemic PAH (pressures: pulmonary artery (PA), 119/57/85 mmHg; ascending aorta (AAO), 94/43/63 mmHg; mean pulmonary arterial pressure (mPAP)/mean systemic arterial pressure (mSAP) ratio, 1.35), severely elevated pulmonary vascular resistance (PVRi, 21 WU × m²; PVR/SVR ratio, 1.2), lack of acute vasoreactivity (AVT) and normal cardiac index (Qsi, 3.6 l min^−1^ m^−2^). Accordingly, at diagnosis, the patient reached ‘higher-risk’ stratification, with a European Pediatric PVD Network (EPPVDN) higher risk score of up to 0.8 (12/15) and lower risk score down to 0 (Supplementary Fig. 1). The electrocardiogram was consistent with tachycardic sinus rhythm, right atrial dilation, RV hypertrophy and strain (Supplementary Fig. 2). Chest X-ray and computed tomography (CT) showed severe dilation of the RV and PAs but normal lung parenchyma and no evidence for thrombi or veno-occlusive disease (Supplementary Figs. 3 and 4). Pulmonary angiograms demonstrated an abnormal peripheral pulmonary vascular pattern characterized by very prominent arterial tortuosity and haziness of the contrast dye in the peripheral pulmonary circulation; the latter may represent diffuse (pre)capillary telangiectasia and/or very small arterio-venous malformations throughout (Supplementary Fig. 5 and Supplementary Video 1a,b).

The patient was found to have a heterozygous missense mutation in the *ACVRL1* gene (c.1451 G>A, p.(Arg484Gln)). This variant occurred de novo and has been previously reported in seven patients with either isolated PAH or PAH plus HHT^11–13^, qualifying her to have heritable PAH and HHT type 2.

After diagnosis (CATH #0), the patient was started on dual oral combination therapy (sildenafil and bosentan) and referred to Hannover Medical School for lung transplant evaluation^14,15^. After limited response to initial dual oral therapy, the PAH-targeted pharmacotherapy was modified to include oral sildenafil, macitentan, spironolactone and inhalative iloprost. Because the systemic blood pressure remained low (systolic, 80 mmHg), we did not start intravenous prostacyclin analogs. Under these measures, the patient was clinically stabilized, and the WHO functional class and 6MWD improved (Supplementary Table 1).

Nevertheless, echocardiographic variables of right ventricle–left ventricle (RV–LV) interaction and LV underfilling (RV/LV end-systolic ratio, LV end-systolic eccentricity index) and pulmonary artery acceleration time (an inverse surrogate of PAH severity) were still greatly abnormal. Global and longitudinal systolic RV function was reduced. Owing to the grim prognosis, allogenic HUCMSC-derived therapy (consented compassionate use) was pursued.

**Fig. 1:**
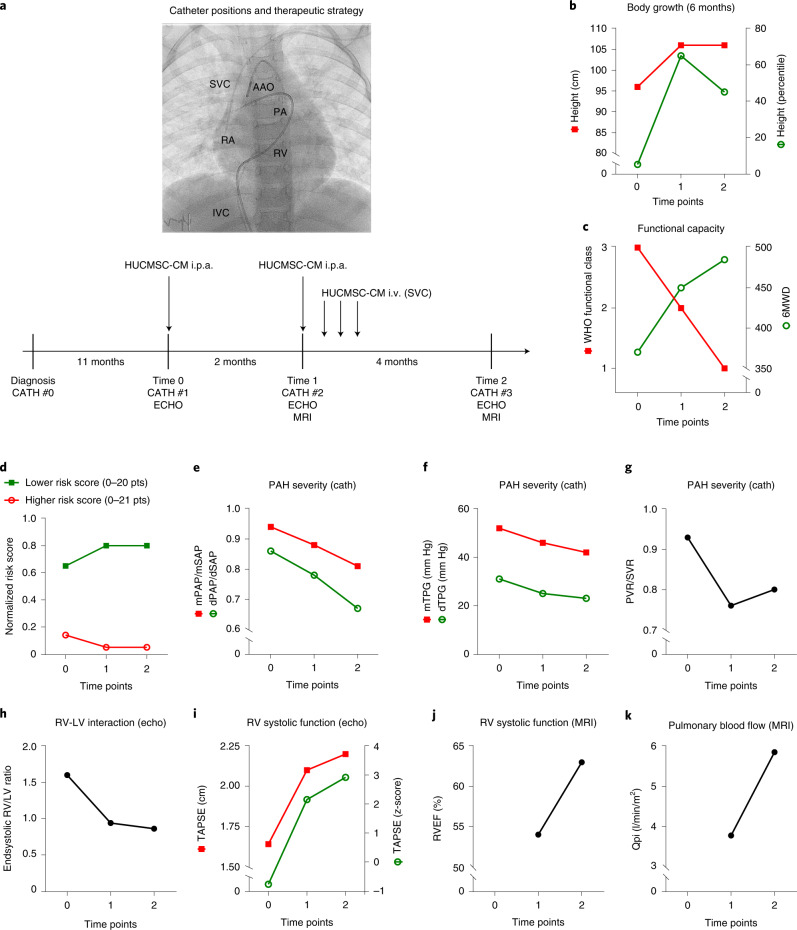
Treatment with HUCMSC-CM results in improvement of growth, functional capacity, risk scores and multiple hemodynamic variables. **a**, Four catheterizations (CATH #0, #1, #2 and #3) were performed spaced 11 months, 2 months and 4 months apart (shown in the timeline as vertical bars). The PAH medication was not changed within the 6 months before CATH #1 (Time 0, at our institution), when, for the first time, HUCMSC-CM was administered in the PAs. The PAH medication was also not changed thereafter. During CATH #1 (Time 0) and CATH #2 (Time 1), after full invasive hemodynamic assessment, 200 ml of HUCMSC-CM was infused in the PAs over 1 hour (100 ml into the right PA over 30 minutes; 100 ml into the left PA over 30 minutes). In the week after CATH #2 (Time 1), 200 ml of HUCMSC-CM was infused via a central venous line on days 1, 2 and 3 after CATH #2. Improvements in body growth (**b**), functional capacity (**c**), EPPVDN risk scores (**d**) and key morphological and hemodynamic data (**e**–**k**) as assessed by cardiac catheterization, echocardiography and cardiac magnetic resonance imaging were generated at the time points specified above (Times 0–2). See Methods for methodological details. cath, right and left heart catheterization; dTPG, diastolic transpulmonary pressure gradient; echo, transthoracic echocardiography; MRI, cardiac magnetic resonance imaging; mTPG, mean transpulmonary pressure gradient; Qpi, pulmonary blood flow index; SVR, systemic vascular resistance. Source data

To this end, we isolated HUCMSCs^16^ from the patientʼs younger sibling’s umbilical cord and collected CM (Methods). The PAH patient received a series of five non-GMP-certified, allogenic HUCMSC-CM infusions over 6 months during two hospital stays (Time 0 and Time 1) via an intrapulmonary arterial catheter and a central venous catheter. All HUCMSC-CM infusions were tolerated very well. Serum CRP and IL-6 levels remained normal, and the patient did not receive any antibiotic, anti-allergic or anti-inflammatory medication. Post-infusion monitoring was at a minimum 24 hours, followed by close outpatient care every 1–6 weeks. Clinical status and invasive hemodynamics were assessed at baseline (Time 0, CATH #1), after 2 months (Time 1, CATH #2) and after 6 months (Time 2, CATH #3) (Fig. 1a).

In the interval between diagnosis and the start of therapeutic HUCMSC-CM intervention, there was essentially no weight gain and no growth (height) in 12 months. After the first HUCMSC-CM infusion (Fig. 1a), the patient started to grow: +10 cm length in 3 months (gain from the 5th to the 65th percentile; Fig. 1b). Moreover, cardiopulmonary exercise capacity greatly increased, as judged by WHO functional class (from 3 to 1) and 6MWD (from 370 m to 485 m; Fig. 1c). The girl is now 6 years old and doing very well, without any limitations in exercise capacity.

Consistent with the clinical improvements, the improved EPPVDN risk scores (Fig. 1d), cardiac catheterization (Fig. 1e–g) and echocardiography (Fig. 1h,i) data confirmed the beneficial effect of HUCMSC-CM treatment: PA pressure (PAP) and PAP gradients (Fig. 1e,f) as well as PVR/SVR ratio (Fig. 1g) decreased by 14–26% (Fig. 1e–g and Supplementary Table 1), indicating a marked decrease in PAH severity. In addition, echocardiography demonstrated that RV–LV interaction as judged by normalized end-systolic RV/LV ratio (Fig. 1h) and RV systolic function (TAPSE; Fig. 1i) had greatly improved (Supplementary Fig. 7 and Supplementary Video 2a,b). Normalization of systolic RV function and pulmonary blood flow were confirmed by cardiac magnetic resonance imaging at Time 1 and Time 2 (Fig. 1j,k and Supplementary Table 1).

**Fig. 2:**
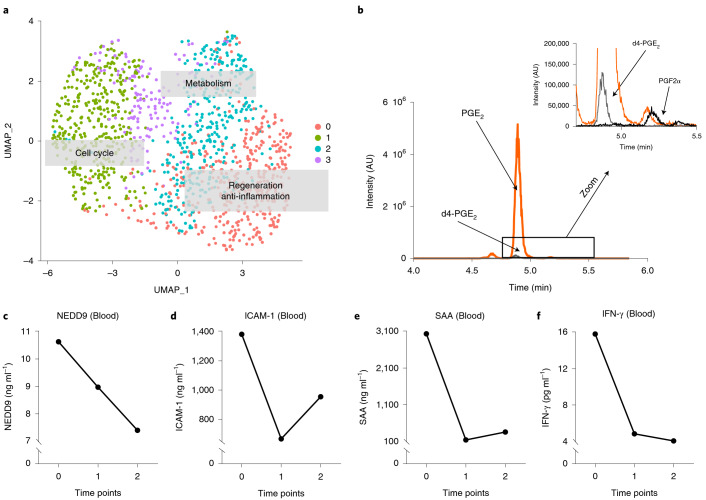
scRNA-seq of HUCMSCs reveals four MSC clusters and a transcriptome enhanced for regeneration, anti-inflammation, cell cycle and metabolism. **a**, Uniform manifold approximation and projection (UMAP) graph of the first two UMAP dimensions shows four clusters of MSCs. Based on the pathway/GO annotation of their marker genes, the functional labels were added to the graph. MSC-like cells were isolated from the human umbilical cord (Wharton’s jelly) after delivery of a full-term infant (38-week gestation). After MSC culture, MSCs were harvested in passage 3 to undergo scRNA-seq, as described in the Methods. **b**, LC–MS chromatogram showing the mass spectrometric traces of PGE_2_, PGF_2α_ and the internal standard d4-PGE_2_. **c**–**f**, EDTA plasma concentrations of NEDD9, ICAM-1, SAA and IFN-γ (mean of concentrations near-simultaneously measured in the SVC, IVC and RA). AU, arbitrary units. Source data

To explore the possible mechanisms of HUCMSC-derived therapy, we performed single-cell RNA sequencing (scRNA-seq) of the subcultured MSCs, which were used to harvest CM for the compassionate use treatment (Fig. 2a), mass spectrometry of MSC-CM (Fig. 2b) and protein expression assays of the patient’s blood plasma collected at different time points (Fig. 2c–f). scRNA-seq of the subcultured HUCMSCs identified four functionally different cell subpopulations (clusters). The four MSC subpopulations are visualized in Fig. 2a with functional labels, based on the pathway/Gene Ontology (GO) annotation of their marker genes (clusters 0–3; Fig. 2a, Supplementary Table 2 and Supplementary Data Table 1). Supplementary Fig. 8 shows an expression heat map of three sets of upregulated genes whose expression separates the cells into the subpopulations (top ten per cluster shown; no upregulated genes for cluster 3). Particularly, cell cluster 0 enhanced the transcriptome for regeneration and anti-inflammation and likely secretes molecules whose paracrine effects provide beneficial effects on right heart–pulmonary circulation.

Based on previous preclinical stem cell studies^17–19^, we hypothesized that boosted prostaglandin E2 (PGE_2_) production may be a major regenerative and immunomodulatory component^20^ in HUCMSC-CM. Indeed, the scRNA-seq data showed that the genes encoding two PGE_2_ synthesis enzymes (*PTGES2* and *PTGES3*), as well as *PTGS2* (*COX2*, a gene involved in conversion of arachidonic acid (AA) into PGH_2_ required for production of PGE_2_), were expressed in most HUCMSCs, as opposed to the enzymes that synthesize PGI_2_ and PGD_2_ (*PTGIS* and *PTGDS* faintly expressed in only a few cells; Supplementary Fig. 9). Consistent with our scRNA-seq data, the analysis of the HUCMSC-CM prostaglandins detected a major PGE_2_ signal but only minimal levels of PGF_2α_ (Fig. 2b) and absent PGD_2_, altogether suggesting that HUCMSC-CM-secreted PGE_2_ may contribute to the beneficial effect of the HUCMSC-CM on our PAH patient.

**Fig. 3:**
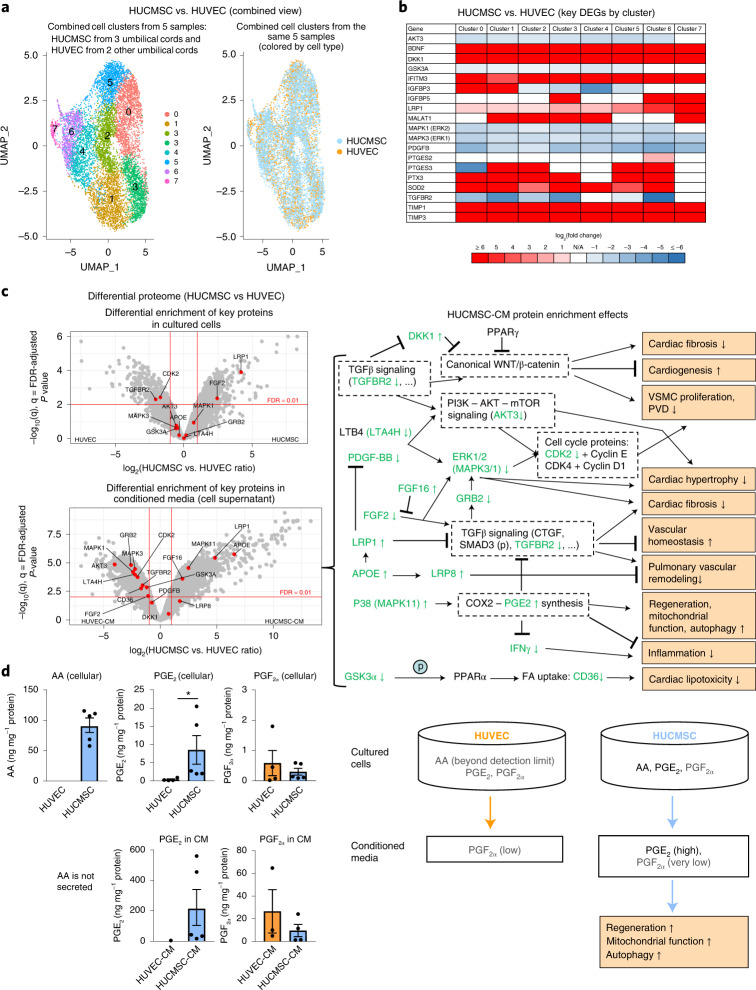
Comprehensive analysis of cultured cells and CM (single-cell transcriptome, proteome and prostaglandins) reveals potential mechanisms explaining regenerative effects of HUCMSC-CM. **a**, Single-cell RNA expression analysis identified eight cell clusters with distinct expression profiles common to both HUCMSC and HUVEC cells. These clusters were used for differential gene expression analysis (HUCMSC versus HUVEC). Sample sizes: HUCMSC, *n* = 3 (passage 6); HUVEC, *n* = 2 (passage 6). All cords were harvested from unrelated donors. **b**, The heat map of the key genes potentially contributing to regenerative effects of HUCMSCs in all eight clusters shown in **a**. Selection of these genes was based on (1) scRNA-seq analysis of the HUCMSCs whose CM was used in treatment of the case; (2) results of the proteomics analysis shown in **c**; and (3) association with synthesis of PGE_2_ (the prostaglandin known for its regenerative capacity). **c**, The volcano plots depict distribution of differentially enriched proteins (HUCMSC, *n* = 5 versus HUVEC, *n* = 4). The key proteins relevant to the regenerative potential are shown as red dots. The proteins above the FDR threshold (<0.01) and passing the effect size threshold (0.5 > HUCMSC versus HUVEC ratio > 2) were considered significant. Concentrations of CM proteins deviated from their levels in cultured cells (in most cases, increasing the degree of differential enrichment). On the right panel, we show potential effects of enrichment of the key proteins in the CM. Proteins shown in green were observed in our data and correspond to the proteins labeled in the volcano plot (↑ = upregulation, ↓ = downregulation, (p) = phosphorylation, bold font indicates FDR-adjusted *P* < 0.01). The right panel depicts likely HUCMSC-CM protein enrichment effects. **d**, LC–MS prostaglandin analysis revealed that the primary difference between HUCMSC (*n* = 5) and HUVEC (*n* = 4) samples was in the significantly higher HUCMSC levels of PGE_2_ (both in cells and CM) and higher levels of AA in HUCMSCs, whereas the HUVEC AA levels were below the detection limit. Because AA is a precursor of PGE_2_, the upregulation of PGE_2_ in HUCMSCs (*P* = 0.0159) is likely associated with the higher HUCMSC AA level. Notably, PGE_2_ greatly contributes to the paracrine regenerative and immunomodulatory effects of MSCs in preclinical in vivo and in vitro studies (for example, PGE_2_-induced suppression of IFN-γ and TGFβ signals). Trace amounts of PGF_2α_ were also detected without significant enrichment in either of the groups. The values in bar plots values are expressed as mean ± SEM; two-tailed Mann–Whitney *U*-test was used (*P < 0.05 and **P < 0.01). DEG, differentially expressed gene. Source data

HUCMSC-derived therapy decreased patient blood plasma markers of vascular (endothelial) fibrosis (NEDD9 (ref. ^21^)), vascular injury (ICAM-1) and inflammation (SAA; IFN-γ) (Fig. 2c–f). These results are consistent with our scRNA-seq data (PGE_2_ synthesis genes) and the subsequent validation in cultured cells and CM (single-cell transcriptome, proteome and prostaglandins) from multiple umbilical cords, described below (Fig. 3).

We expanded our single-cell RNA expression analysis to include HUCMSCs (three umbilical cords) and HUVEC controls (two umbilical cords) and identified eight cell clusters with distinct expression profiles (Fig. 3a).

Based on analysis of differentially expressed genes in these eight clusters (HUCMSC versus HUVEC; Supplementary Data Tables 2–9), we observed that, in general, all eight clusters had expression profiles confirming the beneficial role of HUCMSCs—for example, synthesis of PGE2 and many secreted proteins (for example, DKK1, LRP1 and TGFBR2) known for their role in regenerative pathways (Fig. 3b). Concentrations of CM proteins deviated from their intracellular levels in cultured cells in a way that, in most cases, increased the degree of differential enrichment (Fig. 3c and Supplementary Data Tables 10 and 11).

Analysis of prostaglandins, including the precursor AA (Fig. 3d), demonstrated much higher levels of HUCMSC PGE_2_ (both in cells and CM) and higher levels of AA in HUCMSCs versus HUVECs (HUVEC AA levels were below the detection limit). Because AA is a precursor of PGE_2_, the upregulation of PGE_2_ in HUCMSCs is likely associated with the higher HUCMSC AA level. Trace amounts of PGF_2α_ were also detected without significant enrichment in either of the groups. Taken together, liquid chromatography–mass spectrometry (LC–MS) analysis identified very high levels of PGE_2_ in HUCMSCs and HUCMSC-CM but not in HUVECs or HUVEC-CM (Fig. 3d and Supplementary Data Tables 12 and 13), most likely due to boosted AA-PGE_2_ synthesis in HUCMSCs.

## Discussion

Here we report, to our knowledge, the first-in-human application of HUCMSC-CM to treat severe, progressive PAH in one patient. Serial infusions of HUCMSC-CM resulted in marked clinical and hemodynamic improvement after 6 months and showed no adverse events. The HUCMSC transcriptome (from three umbilical cords, unrelated donors) suggested enhancement of regeneration, mitochondrial function, autophagy and anti-inflammation pathways. Proteomics analysis revealed that the proteins differentially enriched in HUCMSC-CM modulate several key pathways to (1) reduce cardiac fibrosis and hypertrophy, vascular smooth muscle cell (VSMC) proliferation, pulmonary vascular remodeling, inflammation and cardiac lipotoxicity and (2) increase cardiogenesis, vascular homeostasis, regeneration, mitochondrial function and autophagy. Analysis of prostaglandins and AA showed boosted paracrine PGE_2_ signaling derived from cellular AA metabolism in HUCMSCs.

Notably, HUCMSC-derived therapy decreased established blood plasma markers of vascular (endothelial) fibrosis (NEDD9)^21^, vascular injury (ICAM-1) and inflammation (serum amyloid A (SAA) and IFN-γ) in this patient. NEDD9 targets collagen type 3 A1 and promotes endothelial fibrosis in experimental PAH^21^. Moreover, NEDD9 interacts with P-selectin and drives detrimental platelet-endothelial adhesion in the pulmonary circulation^22^. Endothelial *NEDD9* expression is regulated by aldosterone (independently of *TGFβ* signals)^21^ and increased in fibrotic arterioles of patients with PAH^21^. Blood plasma NEDD9 has been shown to be increased in adult PAH by 1.8-fold and to correlate positively with prognostic variables (PVR) and negatively with RV function (right ventricular ejection fraction (RVEF)), exercise capacity (6MWD) and lung-transplant-free survival^23^. Notably, NEDD9 inhibition prevented experimental PAH^21^.

We previously demonstrated that aldosterone, which regulates *NEDD9* in endothelial cells^21^, increases with PAH severity in the blood plasma of adults^24^. Here, HUCMSC-CM treatment, especially the first dose, decreased the circulating vascular injury marker ICAM-1 that is elevated in PAH^24^ and the pro-inflammatory mediators SAA and IFN-γ.

Human MSCs are thought to be immunologically inert, as are cell-free HUCMSC-CM infusions, as shown here. We previously revealed superior regenerative capacity of neonatal tissue-derived MSCs (umbilical cord and placenta) compared to MSCs isolated from adult human tissues (bone marrow, peripheral blood and adipose tissue)^20^. However, long-term tissue engraftment of MSCs has never been demonstrated in preclinical or clinical studies. Accordingly, the major beneficial effects of MSCs are proposed to be of paracrine nature (secretion). MSC-derived extracellular vesicles (EVs)^25^, isolated from CM, have marked efficiency in hyperoxia-induced newborn bronchopulmonary dysplasia in mice^26^ and in *VEGFR2*-blocked/hypoxia-exposed rats with PAH and RV failure^27^. In the latter study, repetitive dosing of bone-marrow-derived MSC-EVs within days was most efficient in reversing PAH^27^. It is likely that EVs contributed to the beneficial effects of our HUCMSC-CM infusions. However, the preclinically used EVs are frequently poorly defined^28^, making inter-study comparisons and GMP-certified therapies for clinical use a difficult task.

We validated our case findings in a subsequent multiple-cord omics analysis of HUCMSCs versus HUVECs and their secretome (CM): at the molecular level, we confirmed upregulation and predicted secretion of the key regeneration/proliferation molecules: *IGFBP3*, *IGFBP5*, *BDNF*, *TIMP1* and *TIMP3* (upregulated in the regenerative cell cluster—that is, cluster 0—of the scRNA-seq results of the treated patient), which were also found as upregulated in most clusters of the integrated scRNA-seq analysis (HUCMSCs versus HUVECs; Fig. 3b) and positively enriched in HUCMSC-CM proteins. Moreover, the results of the proteomics analysis presented in Fig. 3c suggest a variety of mechanisms that likely contribute to improvements of the cardiovascular function in the patient. In brief, our multiple-cord data analysis suggests that (1) downregulation of the *TGFβ* pathway suppresses canonical WNT signaling and, thereby, reduces cardiac fibrosis^29^, promotes cardiogenesis and inhibits VSMC proliferation^30^; (2) downregulation of GSK3α reduces fatty acid (FA) uptake (manifested by CD36 downregulation) and reduces lipotoxicity^31^; (3) suppression of PI3K-AKT-mTOR and ERK1/2 signaling^32^ downregulates VSMC proliferation and cardiac hypertrophy; (4) upregulation of P38 (MAPK11) promotes regenerative effects via induction of COX2-PGE_2_ synthesis^17,33^ (also supported by LC–MS analysis of prostaglandins demonstrating high PGE_2_ levels in HUCMSC but not in HUVEC both in cells and CM (Fig. 3d and Supplementary Data Tables 12 and 13)); (5) upregulation of APOE^34^, LRP1 (ref. ^35^) and FGF16 (ref. ^36^) and downregulation of TGFBR2 and FGF2 (ref. ^36^) suppresses TGFβ signaling, thereby reducing cardiac fibrosis^37^, establishing vascular homeostasis in PAH^8^; (6) suppression of ERK1/2 signaling^32^ inhibits VSMC proliferation; and (7) upregulation of the APOE-LRP8 cascade suppresses pulmonary vascular remodeling^38^.

Our extended multiple-cord LC–MS analysis of prostaglandins and AA confirmed significant upregulation of PGE_2_ (cells and CM) originally identified in the treated case (Fig. 3d). The upregulation of PGE_2_ was also supported by the integrated scRNA-seq data (upregulation of *PTGES2* and *PTGES3* in HUCMSCs) and cell proteomics data, where PTGES was below the detection limit in all HUVEC samples but was present in all HUCMSC samples.

Our multiple-cord omics results underpin the validity of the componential findings and confirm that the way we prepare MSCs and CM is consistent among the different batches—an important point with respect to standardization and reproducibility.

PGE_2_ signaling stimulates stem cells to regenerate damaged tissue^17^, augments mitochondrial function and autophagy and decreases *IFN-γ* and *TGFβ* pathways^19^, which are augmented in PAH^1,7–9^. Of note, these findings have recently funneled into the development of small-molecule 15-prostagalandin dehydrogenase (15-PGDH) inhibitors (SW033291) blocking PGE_2_ degradation^39^; however, clinical trials are pending.

Based on both the very high PGE_2_ levels that we found in the HUCMSC-CM and the induction of three PGE_2_ synthesis enzymes in HUCMSCs, we propose that PGE_2_ is a major beneficial component of HUCMSC-CM, with vasodilatory and regenerative properties in PAH.

We identified a de novo missense mutation in the *ACVRL1* gene in our patient. Intriguingly, this particular *ACVRL1* loss-of-function mutation (c.1451 G>A, p.(Arg484Gln)) has not been found in any patients with HHT in the absence of PAH, underlining the effect of this single-nucleotide variant on pulmonary vascular development and homeostasis.

Our main intent was to report on the impressive improvements in the treated child and the likely mechanisms unraveled by our multi-omics analysis of the case and the available umbilical cord samples. Here, we demonstrate safety and efficacy of MSC-derived therapy, but only in one patient. Additional limitations of our study include a relatively small number of validation cases (three umbilical cords). Further studies are needed for rigorous validation of our findings.

In conclusion, serial infusions of HUCMSC-CM led to marked clinical and hemodynamic improvement in a young patient with severe PAH. We suggest that HUCMSC-derived therapy has the potential to become an efficient treatment for the most severe forms of clinical PAH. F﻿urther prospective clinical studies are warranted to explore the benefits of HUCMSC-derived therapy for PAH.

## Methods

### Isolation of HUCMSC and HUVEC, and preparation of cell-derived CM

#### Isolation, culture and characterization of primary HUCMSC and HUVEC

MSC-like cells were isolated from the human umbilical cord (Wharton’s jelly) after delivery of a full-term (38-week gestation) infant (here: younger sister, by caesarean section; Figs. 1 and 2) and from additional human umbilical cords (Fig. 3). The cells were cultivated by explant culture in MSC growth medium^40^. In brief, umbilical cord tissue was washed several times with PBS to remove blood cells, cut into approximately 1.5-cm^3^-large pieces and incubated in MSC growth medium (αMEM, Invitrogen) supplemented with 15% of allogeneic human AB-serum (HS), 100 U ml^−1^ of penicillin, 100 mg ml^−1^ of streptomycin and 2 mM L-glutamine at 37 °C in a humidified atmosphere with 5% CO_2_. The explant culture was performed for 15 days. The outgrowth of an adherent-enriched MSC population was harvested by accutase (Capricorn Scientific) treatment according to the manufacturer’s protocol for 5 minutes at 37 °C. The cells were centrifuged at 320*g* for 5 minutes, resuspended in MSC culture medium (αMEM) supplemented with 10% of HS, 100 U ml^−1^ of penicillin, 100 mg ml^−1^ of streptomycin and 2 mM L-glutamine at 37 °C in a humidified atmosphere with 5% CO_2_ and cultured at a density of 4,000 cells per cm^2^. Harvesting and subculture into corresponding passages was performed after treatment with accutase (Capricorn Scientific) at 37 °C for 3 minutes.

Continuously proliferating MSCs were harvested and analyzed for cell cycle progression and cell surface marker expression by flow cytometry^40^. Besides detectable G1, S and G2/M phases, the presence of CD73, CD90, and CD105 with concomitant absence of CD14, CD31, CD34, CD45 and HLA-DR was tested by FACS analysis according to the suggestion by the International Society for Cellular Therapy as one of the minimal criteria for MSC characterization^41^. HUVECs were purchased from PromoCell (Cat#C-12200, Heidelberg, Germany), subcultured until P3 or P6 (scRNA-seq), according to the manufacturer’s instructions (PromoCell Instruction Manual). For proteome and LC-MS analyses, CM from HUCMSCs and HUVECs was collected after 3 washing steps in appropriate serum-free medium and incubation of the cultures in corresponding serum-free medium for 36 h, followed by centrifugation (3,185g for 10 minutes).

### Preparation of cell-derived CM from HUCMSC for intravascular infusion

After MSC culture in passages 2 and 3 in subconfluent growth phase, serum-free supernatant was harvested as CM after 36 hours, centrifuged (3.185*g* for 10 minutes), negatively tested for bacterial and mycoplasma contamination and cryo-preserved at −80 °C. The night before injection (about 12–14 hours), the HUCMSC-CM was gently thawed at 4 °C and then pre-warmed to room temperature. For the HUCMSC-CM infusions, certain filters were used: Time 0, CATH #1 (i.p.a): dose 1, 200 ml of CM, Sterifix 0.2-µm filter (B. Braun, 4099303); Time 1, CATH #2 dose 2, i.p.a.: 200 ml of CM, transfusion filter 200 µm (B. Braun, 8270066SP). Time 1, dose 3, SVC; 200 ml of CM, transfusion filter 200 µm (B. Braun, 8270066SP). Time 1, dose 4, SVC: 200 ml of CM, Minisart 0.2-µm filter (Sartorius/Th. Geyer, 90491011), transfusion filter 200 µm (B. Braun). Time 1, dose 5, SVC: 200 ml of CM, Minisart 0.2 µm filter (Sartorius/Th. Geyer, 90491011), transfusion filter 200 µm (B. Braun). HUCMSC-CM doses 2–5 were applied at four consecutive days, respectively.

### Cardiac catheterizations and infusions of HUCMSC-CM

After diagnosis (CATH #0), all subsequent cardiac catheterizations were performed at Hannover Medical School in December 2019 (Time 0, CATH #1), February 2020 (Time 1, Cath #2) and May 2020 (Time 2, CATH #3) (Fig. 1a and Supplementary Table 1). The PAH-targeted medication was not changed in the 6 months before CATH #1, when, for the first time, HUCMSC-CM was administered in the PAs, and not changed thereafter.

During CATH #1 and CATH #2, after full invasive hemodynamic assessment, 200 ml of HUCMSC-CM was infused in the PAs (i.p.a.) over of 1 hour—that is 100 ml into the right pulmonary artery (RPA) over 30 minutes and 100 ml into the left pulmonary artery (LPA) over 30 minutes. In the week after CATH #2, 200 ml of HUCMSC-CM was infused via a central venous line over 60 minutes on days 1, 2 and 3 after CATH #2 (Fig. 1a and Supplementary Fig. 6).

### Microbiological and immunological testing of HUCMSC, HUCMSC-CM, the recipient (patient), donor (sister) and mother

#### HUCMSC/HUCMSC-CM (cell culture supernatant)

Culture 10–14 days negative for bacterial growth (aerobic and anaerobic)

#### Donor

HLA types:

DNA types HLA-1: A*30, A*68, B*13, B*18, C*06, C*12

DNA types HLA-2: DRB3*pos., DQB1*03

HBs-antigen negative, anti-HBC negative, anti-HBS 28 IU l^−1^

Anti-HCV negative, CMV-IgM negative, CMV-IgG 606 U ml^−1^

EBV-IgG 571 E ml^−1^, VCA IgM negative, EBNA1-IgG 141 E ml^−1^

Toxoplasma screening test negative

Treponema pallidum IA-test negative

HIV-AK1/2, p24-Ag negative

#### Recipient

Blood type 0 Rh positive, CcDD.Ee, K-, irregular RBC antibody negative

HLA types:

DNA types HLA-1: A*24, A*68, B*13, B*18, C*06, C*12

DNA types HLA-2: DRB1*07, DRB1*11, DRB3*pos., DRB4*pos., DQB1*02, DQB1*03

GvH constellation (MSC transplant versus patient): A*24, C*04, DRB1*07, DQB1*02

HvG constellation (patient against MSC transplant): A*68, C*06

Mycoplasma IgM negative, mycoplasma IgG negative

### Curaçao’s diagnostic criteria for HHT (Osler–Weber–Rendu syndrome), according to international guidelines (2000 and 2020)^42,43^

These criteria include

1. recurrent and spontaneous epistaxis;
2. the presence of multiple mucocutaneous telangiectasias (characteristic sites: lips, oral cavity, fingers and nose);
3. visceral localization of lesions (such as gastrointestinal telangiectasia and pulmonary, hepatic, cerebral or spinal arteriovenous malformations);
4. an affected first-degree family member with HHT according to these criteria. When an individual shows three or more criteria, they are considered to have HHT; when they meet two criteria, the diagnosis is possible; and with one criterion or none, HHT is considered unlikely using these criteria. A diagnosis of HHT is considered ‘definite’ if three or more Curaçao criteria are present, ‘possible or suspected’ if two criteria are present and ‘unlikely’ if one criterion or none is present.

### Single-cell sequencing of MSC and HUVEC cell samples

Library preparation for single-cell mRNA sequencing analysis was performed according to the Chromium NextGEM Single Cell 3′ Reagent Kit vervion 3.1 User Guide (Manual Part Number CG000204 Rev B, 10x Genomics). A two-fold excess of cells was loaded to the 10x controller in the specified volume to reach a target number of 1,500 cells per sample. Equal molar proportions of eight generated libraries were pooled accordingly, denatured with NaOH and finally diluted to 1.8 pM according to the Denature and Dilute Libraries Guide (document 15048776 v02, Illumina). Next, 1.3 ml of denatured pool was sequenced on an Illumina NextSeq 550 sequencer using one High Output Flowcell for 75 cycles and 400 million clusters (20024906, Illumina). The proprietary 10x Genomics CellRanger pipeline (version 4.0.0) was used with default parameters except for the setting of expected cells (–expect-cells 1500). CellRanger was used to align read data to the human reference genome provided by 10x Genomics (refdata-gex-GRCh38-2020-A) using the STAR aligner. Mean number of reads per cell ranged from 29,916 to 42,827 across all samples. Median number of genes per cell ranged from 3,578 to 4,465 across all samples.

#### ELISA

EDTA blood was collected during cardiac catheterization from the superior vena cava (SVC). The blood samples were immediately centrifuged for 10 minutes at 1,300*g*. Plasma was aliquoted and stored at −80 °C. Plasma samples were diluted 1:3 with Sample Diluent, and plasma neural precursor cell expressed developmentally downregulated protein 9 (NEDD9) concentrations were determined according to the manufacturer’s instructions (Aviva Systems Biology, OKEH02459, Lot KE0777). In brief, 100 ﻿µl of standards, diluted samples and blank were added into the wells of the NEDD9 microplate and incubated at 37 °C for 2 hours. Liquid was discarded, and 100 ﻿µl of biotinylated NEDD9 Detector Antibody was added to each well and incubated at 37 °C for 60 minutes. Liquid was removed, and the microplate was washed with wash buffer. Then, 100 ﻿µl of Avidin-HRP Conjugate was added to each well and incubated at 37 °C for 60 minutes, followed by another washing step. Next, 50 ﻿µl of TMB Substrate was added and incubated at 37 °C in the dark for 15 minutes. Finally, 50 ﻿µl of Stop Solution was added to each well, and the absorbance was read at 450 nm with a wavelength correction of 570 nm.

Plasma ICAM-1 (sample dilution 1:1,000), SAA (sample dilution 1:1,000) and IFN-γ (sample dilution 1:2) concentrations were measured by applying Meso Scale Discovery’s Multi-Array technology, according to the manufacturer’s instructions. ICAM-1 and SAA were measured within Vascular Injury Panel (K15198D-1), and IFN-γ was detected within Proinflammatory Panel (K15049D-1), both according to the manufacturer’s instructions. In brief, the plates were washed three times with 150 ﻿µl per well of wash buffer, and 25 ﻿µl of diluted sample, calibrator or control was added per well (for ICAM-1 and SAA) or 50 ﻿µl of diluted sample, calibrator or control for IFN-γ. The plates were then incubated at room temperature for 2 hours with shaking. After incubation, plates were washed three times with 150 µl per well of wash buffer; 25 ﻿µl of detection antibody was added to each well; and the plate was incubated for an additional 1 hour (for ICAM-1 and SAA) or 2 hours for IFN-γ with shaking at room temperature. Finally, the plates were washed three times with 150 µl per well of wash buffer, and 150 µl of 1× read buffer was added per well (for ICAM-1 and SAA) or 2× read buffer for IFN-γ. Signal intensities were detected and analyzed with a MESO QuickPlex SQ 120 instrument and Discovery Workbench software, version 4.0 (Meso Scale Discovery). The average protein concentrations from the superior vena cava (SVC) and inferior vena cava (IVC) and the right atrium (RA) are reported.

### Label-free quantitative discovery proteomics

#### Sample preparation for proteomics

Protein was extracted from HUVECs and HUCMSCs, and DNA was sheared in 40 µl of lysis buffer (1% SDS, 0.1 M ABC, 1.25× PIC) in AFA-TUBE TPX Strips on a Covaris LE220Rsc by focused ultrasonication (PIP 450 W, DF 25%, CPB 200, two repeats, 300-second pulse, 20 °C). Samples were cleared from debris (2,500*g* for 5 minutes) and protein quantified (Pierce BCA, 23225). Samples of 30 µg of cellular protein were filled to 50 µl with lysis buffer, and 16.6 μl of reduction and alkylation buffer (40 mM TCEP, 160 mM CAA, 200 mM ABC) was added. Secreted proteins in the CM (200 µl) were concentrated (overnight lyophilization) and reconstituted in 40 µl of 10 mM TCEP and 40 mM CAA. Cellular and secreted proteins were prepared using the SP3 protocol with single-step reduction and alkylation^44^ on a Beckman Biomek i7 workstation. Samples were incubated for 5 minutes at 95 °C and cooled to room temperature. Proteins were bound to 250-μg paramagnetic beads (1:1 ratio of of hydrophilic/hydrophobic beads) by adding acetonitrile (ACN) to 50% for cellular proteins or 70% for secreted proteins, respectively. Samples were washed twice with 80% ethanol and once with 100% ACN, before reconstitution in 35 μl of 100 mM ABC. Digestion was completed overnight at 37 °C using a trypsin/LysC enzyme mix (Promega) at a ratio of protein:enzyme of 50:1 for cellular proteins and 250 ng for secreted proteins, respectively. The reaction was stopped with FA (0.1%), and the peptides were stored at −80 °C until analysis without further conditioning or cleanup.

#### Proteome analysis by data-independent acquisition LC–MS

The amount of injected tryptic digest was set to 40 ng, the available material for the lowest concentrated sample. Peptides were resolved on a 25-cm Aurora Series with emitter column (CSI, 25 cm × 75 µm ID, 1.6-µm C18, IonOpticks, installed in the nano-electrospray source (CaptiveSpray source, Bruker Daltonics) at 50 °C using an UltiMate 3000 (Thermo Fisher Scientific Dionex) coupled with TIMS quadrupole time-of-flight instrument (timsTOF Pro2, Bruker Daltonics) and measured in diaPASEF mode. The mobile phases water/0.1% FA and ACN/0.1% FA (A and B, respectively) were applied in the linear gradients starting from 2% B and increasing to 17% in 87 minutes, followed by an increase to 25% B in 93 minutes, 37% B in 98 minutes and 80% B in 99–104 minutes, and the column was equilibrated in 2% B by next 15 minutes. For calibration of ion mobility dimension, three ions of Agilent ESI-Low Tuning Mix ions were selected (*m*/*z* [Th], 1/*K*0 [Th]: 622.0289, 0.9848; 922.0097, 1.1895; 1221.9906, 1.3820). The diaPASEF windows scheme ranged in dimension *m*/*z* from 396 to 1,103 Th and in dimension 1/*K*0 0.7–1.3 Vs cm- 2, with 59 ×12 Th windows). All measurements were done in Low Sample Amount Mode with Ramp Time of 166 ms.

#### Protein identification and quantification

The raw data were processed using DIA-NN 1.8 (ref. ^45^) with the ion mobility module for diaPASEF (Demichev et al., https://www.biorxiv.org/content/10.1101/2021.03.08.434385v1 (2021)). MS2 and MS1 mass accuracies were both set to 10 p.p.m., and scan window size was set to 10. DIA-NN was run in library-free mode with standard settings (fasta digest and deep-learning-based spectra, RT and IMs prediction) using the UniProt human reference proteome annotations (UP000005640_9606, downloaded on 20 December 2019)^46^ and the match-between-runs (MBR) option.

### LC–MS of cells and CM

Samples were spiked with internal standards to a final concentration of 1.0 ng ml^−1^ and prepared using solid-phase extraction as described previously^47^, and 200 µl of CM samples was used. Medium samples were reconstituted in 200 µl of 40% MeOH and cell pellet samples in 100 µl of 40% MeOH. LC–MS analysis was carried out using two LC-30AD pumps, a SIL-30AC autosampler and a CTO-20AC column oven (all Shimadzu). The autosampler was held at 6 °C. Forty microliters was injected, and separation was accomplished on a Kinetex C18 column (Phenomenex, 50 ×2.1 mm, 1.7 µm) using a gradient of 0.01% acetic acid (Fluka) in water (Honeywell-Riedel de Haën; A) and 0.01% acetic acid in MeOH (B). The oven was held at 50 °C. The gradient was as follows: 0.0–1.0 minutes constant at 30% B, 1.0–1.1 minutes linear increase to 45% B, 1.1–2.0 minutes linear increase to 53.5% B, 2.0–4.4 minutes linear increase to 55.5% B, 4.0–7.0 minute linear increase to 90% B, 7.0–7.1 minutes linear increase to 100% B, 7.1–9.0 minutes constant at 100% B, 9.0–9.5 minutes linear decrease to 30% B, 9.5–11.5 minutes constant at 30% B. Detection was achieved on a Qtrap 6500 (Sciex Nieuwerkerk a/d IJssel) equipped with a ESI source. The MS was operated in negative scheduled MRM mode. The needle voltage of the source was set at −4,500 V, the drying temperature at 450 °C, ion source gas 1 and 2 (both air) at, respectively, 40 p.s.i. and 30 p.s.i. and the nebulizer gas (nitrogen) at 30 p.s.i. The entrance potential was set to 10 V and the collision gas flow to ‘medium’. Detailed settings can be found elsewhere^48^. Calibration ranges and functions are given in Supplementary Tables 6 and 7. All calibration lines were weighed 1/x^2^. Results were expressed as ng ml^−1^ or ng prostaglandin per mg protein (cell pellet). Protein was quantified using the BCA assay according to the manufacturer’s instructions.

To confirm the regenerative, MSC-derived effect of PGE_2_, we included only a small panel of selected prostaglandins (PGD_2_, PGF_2α_, PGE_2_, 8-iso-PGE_2_ and 8-iso-PGF_2α_) and their precursor AA in the LC–MS/MS analysis.

### Statistical analysis

scRNA-seq data analysis was performed using the Seurat R package (version 4.0.2). Two types of scRNA-seq analysis were performed. (1) The HUCMSCs that generated the CM used for treating the reported case (sample ID: HUCMSC1_P3_female; cell number: 1418) were analyzed for the presence of cell clusters with regeneration potential. After the standard analysis steps in Seurat (as outlined in the manual), including regressing out the cell cycle effects, we performed unsupervised clustering of the single-cell data. (2) Integrated scRNA-seq analysis of three other HUCMSC samples (sample IDs: HUCMSC2_P6_male, HUCMSC3_P6_male, HUCMSC4_P6_male; respective cell numbers: 1317, 1982 and 3203) and two HUVEC samples (sample IDs: HUVEC1_P6_male, HUVEC2_P6_male; respective cell numbers: 1316 and 2397) was performed as outlined in the Seurat tutorial (https://satijalab.org/seurat/archive/v3.1/immune_alignment.html). Before this analysis, standard filtration was performed. However, given that the difference in proliferation rates between HUCMSCs and HUVECs may be biologically relevant, we chose not to regress out the cell cycle effects. Batch effect correction and normalization was performed using the SCTransform function from the SCTransform R package (version 0.3.3). Percentage of mitochondrial genes and sample IDs were used as variables to regress out in SCTransform. Upon batch effect correction, sample percentages per cluster ranged as follows: cluster 0 (16.87–21.67%), cluster 1 (18.47–20.9%), cluster 2 (15.02–25.17%), cluster 3 (18.53–21.01%), cluster 4 (13.19–26.44%), cluster 5 (16.14–23.87%), cluster 6 (13.03–27.58%) and cluster 7 (0–40.44%). Seurat’s function SelectIntegrationFeatures with the number of features set to 3,000 was used for feature selection. Seurat’s ElbowPlot function was used to estimate the number of meaningful dimensions. The marker genes used for definition of the cell clusters or differentially expressed genes (false discovery rate (FDR)-adjusted *P* < 0.05) from the integrated analysis (HUCMSC versus HUVEC) were analyzed for GO and pathway overrepresentation using the online tool Enrichr (https://maayanlab.cloud/Enrichr/). Differentially expressed genes were identified using the FindMarkers function with default parameters from the Seurat package.

Proteomics analysis was performed using the DEP R package (version 1.12.0) with default parameters. In addition, we used GSEA (version 4.1.0) with default parameters to perform gene enrichment set analysis of differentially expressed genes and differentially enriched proteins. Prostaglandin (LC–MS) results were analyzed using GraphPad Prism (version 7). Two-tailed Mann–Whitney *U*-tests were used, because normality could not be checked due to small sample sizes.

### Ethical considerations

The use of primary human MSCs after explant culture from umbilical cord tissue was approved by the Ethics Committee of Hannover Medical School (Research Obstetric Biobank; institutional review board number 1303-2012/update 2020). The caregivers (parents) of the treated patient gave written informed consent for compassionate use of therapy, bioanalysis and publication of the data. This report is in line with CARE guidelines.

### Reporting summary

Further information on research design is available in the Nature Research Reporting Summary linked to this article.

### Supplementary information

Supplementary Information Supplementary Figs. 1 –9 and Supplementary Tables 1 and 2

Reporting Summary

Supplementary Video 1 **Movies S1 A. Pulmonary artery angiograms in the left pulmonary artery**. (**A**: posterior-anterior, **B**: lateral). Contrast was injected via a pigtail catheter (4F, Terumo) placed **left lower lobe pulmonary artery**. There is no prominent narrowing at the lobar and segmental branches, and no obvious pruning of the subsegmental branches. The peripheral pulmonary vascular pattern appears grossly abnormal: very prominent tortuosity of the peripheral pulmonary arteries is shown. The prominent haziness of the contrast dye in the peripheral pulmonary circulation may represent diffuse (pre)capillary telangiectasia and/or very small arterio-venous malformations throughout. No obvious large pulmonary arterio-venous malformations (AVMs) are visualized (SpO_2_ > 95%). See complete selective pulmonary angiograms in movies S2A, S2B. On pulmonary venous recirculation, contrast returns normally via the regularly sized left lower lobe pulmonary vein to the left atrium. There is no obvious flow of contrast from the left atrium to the right atrium (no significant atrial shunt). See also Extended Data Figure 5 for main and left pulmonary artery angiogram, and Extended Data Figure 6 for catheter positions during HUCMSC-CM infusions

Supplementary Video 2 **Movies S1 B. Pulmonary artery angiograms in the left pulmonary artery**. (**A**: posterior-anterior, **B**: lateral). Contrast was injected via a pigtail catheter (4F, Terumo) placed **left lower lobe pulmonary artery**. There is no prominent narrowing at the lobar and segmental branches, and no obvious pruning of the subsegmental branches. The peripheral pulmonary vascular pattern appears grossly abnormal: very prominent tortuosity of the peripheral pulmonary arteries is shown. The prominent haziness of the contrast dye in the peripheral pulmonary circulation may represent diffuse (pre)capillary telangiectasia and/or very small arterio-venous malformations throughout. No obvious large pulmonary arterio-venous malformations (AVMs) are visualized (SpO_2_ > 95%). See complete selective pulmonary angiograms in movies S2A, S2B. On pulmonary venous recirculation, contrast returns normally via the regularly sized left lower lobe pulmonary vein to the left atrium. There is no obvious flow of contrast from the left atrium to the right atrium (no significant atrial shunt). See also Extended Data Figure 5 for main and left pulmonary artery angiogram, and Extended Data Figure 6 for catheter positions during HUCMSC-CM infusions

Supplementary Video 3 **Movies S2 A, B. Transthoracic echocardiography (B mode): Parasternal short axis view (PSAX) at baseline (Time 0) and 6 months after the start of HUCMSC-CM therapy (Time 0)**. Assessment of the end-systolic curvature of the interventricular septum (IVS) shows in **A** (baseline, time 0) complete flattening of the IVS (D-sign), indicating systemic right ventricular pressure, consistent with a mPAP/mSAP ratio of 0.94 determined on the subsequent day in the cardiac catheterization laboratory. Six months after the start of HUCMSC-CM therapy (**B**, time 2), the IVS curvature in end-systole is not flat but rather convex to the RV, indicating sub-systemic RV and PA pressure, consistent with a mPAP/mSAP ratio of 0.81 determined on the subsequent day in the cardiac catheterization laboratory. See also in the main manuscript Fig. 1E for mPAP/mSAP ratios and Fig. 1H for RV/LV end-systolic ratios over time. The according still frames, taken in end-systole, are shown in Extended Data Figure 5

Supplementary Video 4 **Movies S2 A, B. Transthoracic echocardiography (B mode): Parasternal short axis view (PSAX) at baseline (Time 0) and 6 months after the start of HUCMSC-CM therapy (Time 0)**. Assessment of the end-systolic curvature of the interventricular septum (IVS) shows in **A** (baseline, time 0) complete flattening of the IVS (D-sign), indicating systemic right ventricular pressure, consistent with a mPAP/mSAP ratio of 0.94 determined on the subsequent day in the cardiac catheterization laboratory. Six months after the start of HUCMSC-CM therapy (**B**, time 2), the IVS curvature in end-systole is not flat but rather convex to the RV, indicating sub-systemic RV and PA pressure, consistent with a mPAP/mSAP ratio of 0.81 determined on the subsequent day in the cardiac catheterization laboratory. See also in the main manuscript Fig. 1E for mPAP/mSAP ratios and Fig. 1H for RV/LV end-systolic ratios over time. The according still frames, taken in end-systole, are shown in Extended Data Figure 5

Supplementary Data 1 Supplementary Data Tables 1–13

### Source data

Source Data Fig. 1 Statistical Source Data

Source Data Fig. 2 Statistical Source Data

Source Data Fig. 3 Statistical Source Data

## Data Availability

The scRNA-seq data are accessible via the National Center of Biotechnology Information Gene Expression Omnibus (accession ID: GSE199071). We have deposited the raw data for proteomics experiments to PRIDE (EMBL), which is a part of ProteomeXchange (accession ID: PXD032234). The LC–MS prostaglandin data are in the supplementary dataset (Supplementary Data Tables 12 and 13).
