# Supplementary material for: Human umbilical cord mesenchymal stem cell-derived treatment of severe pulmonary arterial hypertension
Source: Nat Cardiovasc Res. 2022 Jun 9;1(6):568–76. doi: 10.1038/s44161-022-00083-z (PMC11358026; doi:10.1038/s44161-022-00083-z)
Supplement: Supplementary file 1 — Supplementary Figs. 1 –9 and Supplementary Tables 1 and 2 [file 44161_2022_83_MOESM1_ESM.pdf]

nature portfolio

https://doi.org/10.1038/s44161-022-00083-z

Supplementary information

Human umbilical cord mesenchymal stem cell-derived treatment of severe pulmonary arterial hypertension

In the format provided by the authors and unedited

SUPPLEMENTARY INFORMATION

Human Umbilical Cord Mesenchymal Stem Cell-Derived Treatment of Severe Pulmonary Arterial Hypertension

Georg Hansmann[1,2] *, Philippe Chouvarine[1,2], Franziska Diekmann[1,2],

Martin Giera[3], Markus Ralser[4], Michael Mülleder[4], Constantin von Kaisenberg[5],

Harald Bertram[1], Ekaterina Legchenko[1,2], Ralf Hass[6]

Author affiliations:

[1] Department of Pediatric Cardiology and Critical Care, Hannover Medical School, Hannover, Germany

[2] European Pediatric Pulmonary Vascular Disease Network, Berlin, Germany

[3] Center for Proteomics and Metabolomics, Leiden University Medical Center (LUMC), Albinusdreef 2, 2333 ZA, Leiden, The Netherlands

[4] Department of Biochemistry, Charité Universitätsmedizin Berlin, Berlin, Germany

[5] Departments of Obstetrics, Gynecology and Reproductive Medicine, Hannover Medical School, Hannover, Germany

[6] Biochemistry and Tumor Biology Lab, Department of Gynecology, Hannover Medical School, Hannover, Germany

Keywords: mesenchymal stem cells – human umbilical cord – pulmonary hypertension – pulmonary vascular disease – right heart failure – treatment

*** Correspondence should be addressed to:**

Prof. Dr. Georg Hansmann, M.D., Ph.D., FESC, FAHA
Department of Pediatric Cardiology and Critical Care
Pulmonary Vascular Research Center
Hannover Medical School
Carl-Neuberg-Str. 1, 30625 Hannover, Germany
Phone: +49 511 532 9594
Email: georg.hansmann@gmail.com
Twitter: @Hansmann_Lab and @PVD_Network

Table of Contents

LIST OF INVESTIGATORS **3**

ABBREVIATIONS AND ACRONYMS **4**

SUPPLEMENTARY FIGURES **5**

Supplementary Figure 1. The 2019 EPPVDN Risk Score for Pediatric Pulmonary Hypertension 5

Supplementary Figure 2. Electrocardiogram (ECG) at the time of diagnosis 7

Supplementary Figure 3. Chest X-Ray at the time of PAH diagnosis 8

Supplementary Figure 4. Chest Computed Tomography at the time of PAH diagnosis 8

Supplementary Figure 5. Pulmonary Angiogram: Main and Left Pulmonary Artery (MPA, LPA) 9

Supplementary Figure 6. Catheter Positions during HUCMSC-CM Infusions 10

Supplementary Figure 7. Echocardiography Indicates Improved RV-LV-Interaction 6 months after Start of HUCMSC-CM Therapy 11

Supplementary Figure 8. Single-cell RNA Sequencing of HUCMSC reveals four MSC clusters 12

Supplementary Figure 9. Prostaglandin (PGE_2) synthesis enzymes that are ubiquitously expressed in the majority of the MSC cells as opposed to the enzymes that synthesize PGI_2 and PGD_2 13

SUPPLEMENTARY MOVIES **14**

Supplementary Movie 1. Pulmonary artery angiograms in the left pulmonary artery. 14

Supplementary Movie 2. Transthoracic echocardiography (B mode), parasternal short axis view (PSAX) at baseline (Time 0) and 6 months after the start of HUCMSC-CM therapy (Time 0). 14

SUPPLEMEMTARY TABLES **15**

Supplementary Table 1. Characteristics of PAH patient at baseline (Cath #1) and at follow up (Caths #2-3) – extended data set 15

Supplementary Table 2. Functional annotation of the human umbilical cord MSC subpopulations 17

LIST OF INVESTIGATORS

Georg Hansmann M.D., Ph.D.*

Philippe Chouvarine, Ph.D.

Franziska Diekmann

Martin Giera, Ph.D.

Markus Ralser, Ph.D.

Michael Mülleder, Ph.D.

Constantin von Kaisenberg, M.D.

Harald Bertram, M.D.

Ekaterina Legchenko, Ph.D.

Ralf Hass, Ph.D.

*corresponding author

ABBREVIATIONS and ACRONYMS

AVM = arterio-venous malformation
AVT = acute pulmonary vasoreactivity testing
CI = cardiac index
CATH = cardiac catheterization
COL3A1 = collagen type 3 A
DPD = diastolic pressure difference
FPAH/HPAH = familial/heritable pulmonary arterial hypertension
ICAM-1 = intercellular adhesion molecule 1
IFN-γ = interferon gamma
IPAH = idiopathic pulmonary arterial hypertension
LAP = left atrial pressure
LV = left ventricle
NEDD9 = neural precursor cell expressed developmentally down-regulated protein 9
mRAP = mean right atrial pressure
dPAP = diastolic pulmonary artery pressure
mPAP = mean pulmonary artery pressure
sPAP = systolic pulmonary artery pressure
dSAP = diastolic systemic artery pressure (aorta)
mSAP = mean systemic artery pressure (aorta)
sSAP = systolic systemic artery pressure (aorta)
mTPG = mean transpulmonary pressure gradient
dTPG = diastolic transpulmonary pressure gradient (syn. DPG)
iNO = inhaled nitric oxide
NT-proBNP = N-terminal pro B-type natriuretic peptide
PAH = pulmonary arterial hypertension
PAWP = pulmonary artery wedge pressure
PAAT = pulmonary artery acceleration time
PH = pulmonary hypertension
PHVD = pulmonary hypertensive vascular disease
PPHN = persistent pulmonary hypertension of the newborn
PVD = pulmonary vascular disease
PVRi = pulmonary vascular resistance index (PVR indexed to body surface area)
Qpi = pulmonary blood flow index (Qp indexed to body surface area)
Qi = systemic blood flow index (Qp indexed to body surface area), syn. cardiac index
RAP = right atrial pressure
RCT = randomized controlled trial
RV = right ventricle
RVAWD = right ventricular wall diameter (in diastole)
RVEDD = right ventricular end-diastolic diameter (syn. RVIDd)
RV/LV end-systolic ratio = ratios of inner diameters of RV over LV in end-systole
SAA = serum amyloid A protein
S/D ratio = systolic/diastolic duration ratio, CW Doppler flow of tricuspid regurgitation flow
s/p = status post
SVRi = systemic vascular resistance (SVR indexed to body surface area)
TAPSE = tricuspid annular plane systolic excursion
TPG = transpulmonary pressure gradient
TR = tricuspid regurgitation
TRV = tricuspid regurgitation velocity (m/s)
WHO = World Health Organization
WSPH = World Symposium on Pulmonary Hypertension

SUPPLEMENTARY FIGURES

Supplementary Figure 1. The 2019 EPPVDN risk score for pediatric pulmonary hypertension

Patient

....	....	....
**Surname, First Name**	**Date of Birth**	**Patient´s ID**

**Parameter**	**Measured Variable**	**Lower Risk Criteria**		**Higher Risk Criteria**	
**Clinical Presentation**	**Clinical evidence of RV failure** (e.g. exertional dyspnoea, fatigue, dizziness, ankle swelling, epigastric fullness and right upper abdominal discomfort or pain)	no	☐	yes	☐
	**Progression of symptoms**	no	☐	yes	☐
	**Syncope**	no	☐	yes	☐
	**Growth**	Normal (height, BMI)	☐	Failure to thrive	☐
	**WHO functional class**	*I, II	☐	*III, IV	☐
**Laboratory Results**	**Serum NT-proBNP**	*Minimally elevated for age or not elevated	☐	*Greatly elevated for age, i.e. >1200 pg/mL (>1yr old)<br>Rising NT-proBNP level	☐
**Medical Imaging**	**Echocardiography, CMR**	Minimal RA/RV enlargement<br>No RV systolic dysfunction<br>RV/LV endsystolic ratio < 1 (PSAX)<br>TAPSE normal (z > -2)<br>S/D ratio <1.0 (TR jet)<br>PAAT > 100 ms (>1yr old)	☐<br>☐<br>☐<br>☐<br>☐<br>☐	Severe RA/RV enlargement<br>RV systolic dysfunction<br>RV/LV endsystolic ratio >1.5 (PSAX)<br>TAPSE (z < -3)<br>S/D ratio >1.4 (TR jet)<br>PAAT <70 ms (>1yr old)<br>Pericardial effusion	☐<br>☐<br>☐<br>☐<br>☐<br>☐<br>☐
**Cardiac Catheterization**<br>Last CATH study (date):<br>.............<br>(preceding 12 months)	**Invasive Hemodynamics**	*Cardiac index >3.0 l/min/m²<br>*mRAP <10 mm Hg<br>mPAP/mSAP <0.5<br>Acute vasoreactivity +	☐<br>☐<br>☐<br>☐	*Cardiac index <2.5 l/min/m²<br>*mRAP >15 mm Hg<br>mPAP/mSAP >0.75<br>PVRi >15 WU x m²	☐<br>☐<br>☐<br>☐

**Lower Risk**	**Intermediate Risk**	**Higher Risk**
= at least 3 starred (*) lower-risk and no higher-risk criteria (CATH available).<br>or<br>= at least 5 non-starred lower-risk and no higher-risk criteria (CATH not available).	= definitions of lower or higher risk not fulfilled.	= at least 2 starred (*) higher-risk criteria including cardiac index (CATH available).<br>or<br>= greatly elevated NT-proBNP* and at least 5 non-starred higher-risk criteria (CATH not available).
Date:.................. ☐	Date:................ ☐	Date:................ ☐

Supplementary Figure 1: Pediatric pulmonary hypertension - individual risk stratification. The above 2019 EPPVDN risk score sheet for a child with pulmonary hypertension may be used at follow-up in clinics (from Hansmann G et al. J heart Lung Transplant, 2019). While serum NT-proBNP and many of the listed echocardiographic variables have normative reference values (z-scores, range) and have been validated to some extent in children with PH, this is not the case for most invasive hemodynamic criteria. Thus, the risk stratification and combination of criteria in this figure is primarily a consensus within the according task force of these guidelines. Note that changes in PAH medication and/or clinical condition often are associated with changes in hemodynamics. Only cardiac catheterization data from the preceding 12 months should be taken into account. The starred criteria (*) are risk determinants/prognostic variables with high prognostic impact on clinical outcome, based on retrospective analyses of adult PAH study data (WHO FC, NT-proBNP, cardiac index, mRAP), and are counted as 2 points.
Abbreviations: CMR – cardiac magnetic resonance imaging; NT-proBNP – N-terminal pro B-type natriuretic peptide; RV-failure – right ventricular failure; RV – right ventricle; RA – right atrium ; PAAT, pulmonary artery acceleration time; PVRi - pulmonary vascular resistance index; mPAP – mean pulmonary arterial pressure; mRAP – mean right atrial pressure; S/D, systolic/diastolic duration ratio, CW Doppler flow of tricuspid regurgitation flow; TR – tricuspid regurgitation**.**

HOW TO DETERMINE THE EPPVDN RISK SCORE?

• If the patient had a recent cardiac catheterization (within the preceding 12 months), the maximum lower risk score (including 4 * criteria) is 20, and the maximum higher risk score (including 4 * criteria) is 21.

• If the patient has not had any recent cardiac catheterization within the preceding 12 months, the maximum lower risk score (including 2 * criteria) is 14 and the maximum higher risk score (including 2 * criteria) is 15.

• Accordingly, the actual lower risk and higher risk scores can be given as points per max. score (e.g., “8/20 lower risk score for a patient with recent cardiac catheterization data in the preceding 12 months”).

• The EPPVDN risk score is indicated as ratio (actual score per max. score) and as decimal number, e.g. “the max. lower EPPVDN risk score is 10/20 (0.5).

The above 2019 EPPVDN risk score is taken from Hansmann G, Koestenberger M, Alastalo T, Apitz C, Austin E, Bonnet D, Budts W, D’Alto M, Gatzoulis MA, Hasan BS, Kozlik-Feldmann R, Kumar RK, Lammers AE, Latus H, Michel-Behnke I, Miera O, Morrell NW, Pieles G, Quandt D, Sallmon H, Schranz D, Tran-Lundmark K, Tulloh RMR, Warnecke G, Wåhlander H, Weber SC and Zartner P. 2019 Updated Consensus Statement on the Diagnosis and Treatment of Pediatric Pulmonary Hypertension. The European Pediatric Pulmonary Vascular Disease Network (EPPVDN), endorsed by AEPC, ESPR and ISHLT. J Heart Lung Transplant. 2019; DOI: https://doi.org/10.1016/j.healun.2019.06.022 (ref.[50])

We have started validating the above 2019 EPPVDN pediatric PH risk score in: Hansmann G, Meinel, K, Bukova M, Chouvarine P, Wåhlander H, Koestenberger M, on behalf of the European Pediatric Pulmonary Vascular Disease Network (EPPVDN). Selexipag for the treatment of children with pulmonary arterial hypertension: First multicenter experience in drug safety and efficacy. J Heart Lung Transplant 2020; https://doi.org/10.1016/j.healun.2020.03.0 (ref.[51]).

The 2019 EPPVDN pediatric PH risk score is now available as free online web application (https://www.pvdnetwork.org/pedphriskscore/).

Supplementary Figure 2. Electrocardiogram (ECG) at the time of diagnosis

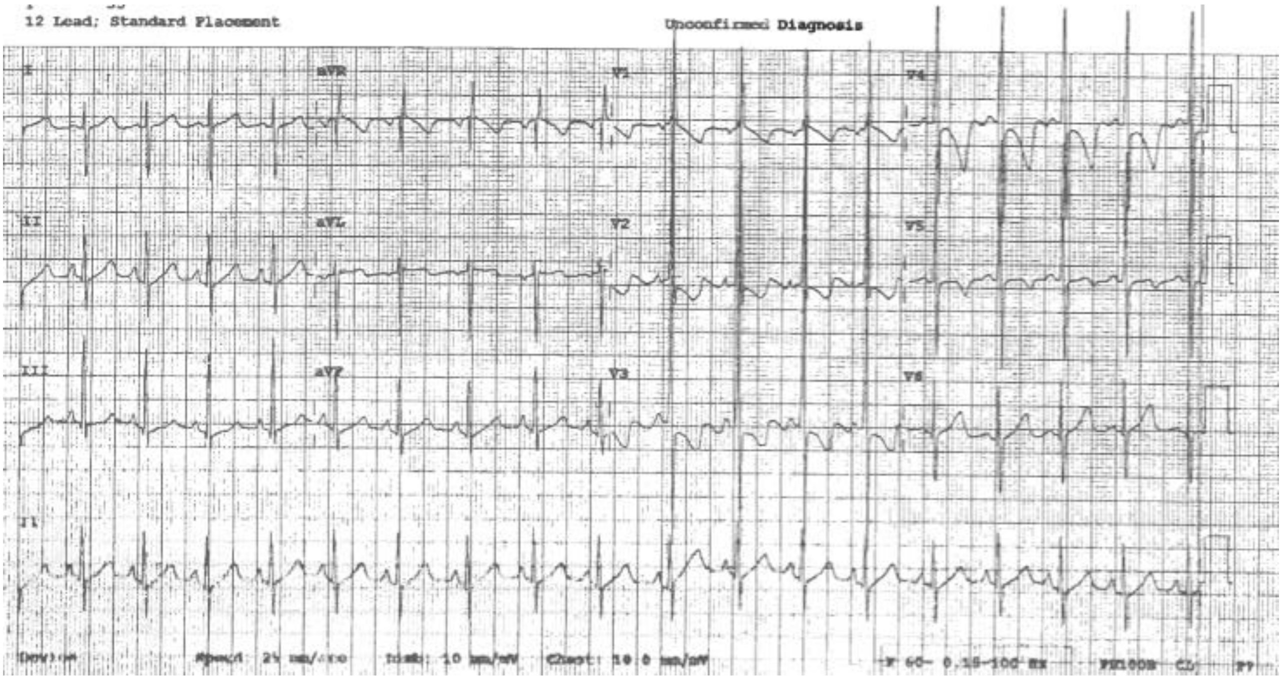

Supplementary Figure 2. Electrocardiogram of the 3-year-old female patient at the time of diagnosis "severe PAH". The ECG was recorded 2 weeks prior to the diagnostic cardiac catheterization (CATH #0) and shows sinus rhythm, tachycardia at rest (109 bpm), right axis deviation, right atrial enlargement, right ventricular hypertrophy, and right precordial strain pattern with descending ST in V1-V4 and T inversions in V1-V5.

Supplementary Figure 3. Chest X-Ray at the time of PAH diagnosis

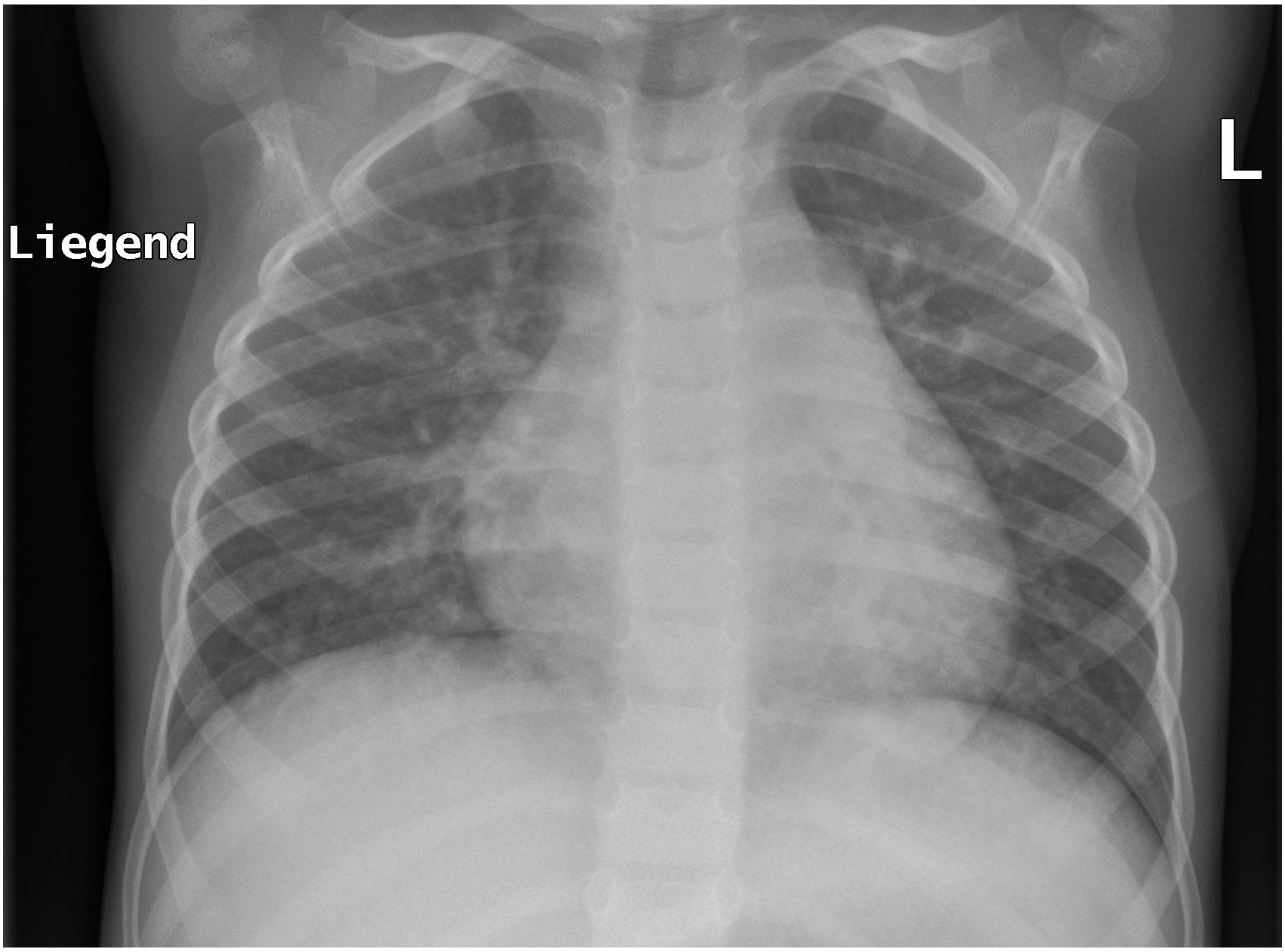

Supplementary Figure 3. ***Chest X-ray (a.p.)*** of the 3-year-old female patient with severe heritable pulmonary arterial hypertension (HPAH) and hereditary hemorrhagic telangiectasia type 2 (HHT; de novo ACVRL1 mutation), at the time of diagnosis, shows cardiomegaly and mild pulmonary hyperperfusion, but no pleural effusions and no infiltrates.

Supplementary Figure 4. Chest computed tomography at the time of PAH diagnosis

A. Chest CT Angiography (Aorta, PA) **B. Chest CT, Lung Window**

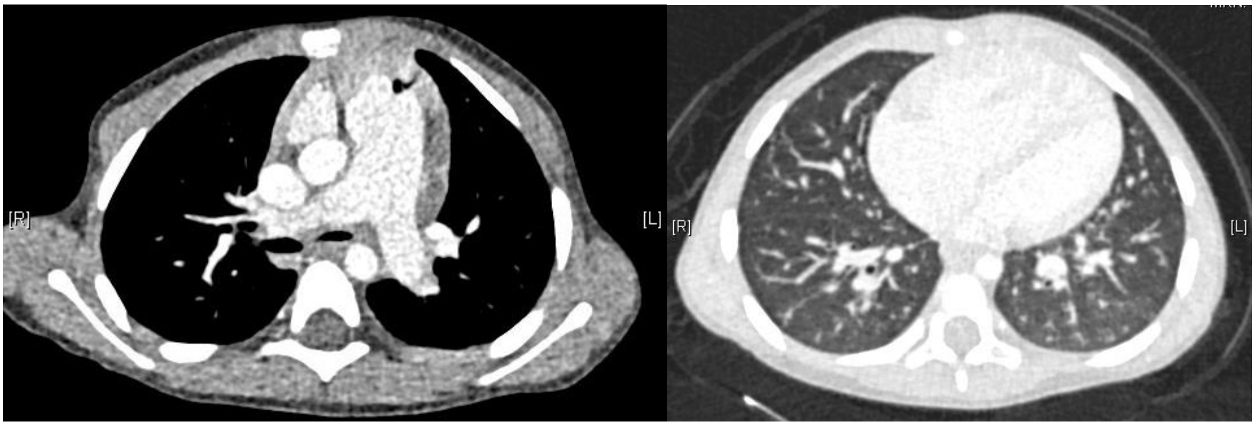

Supplementary Figure 4. ***Chest computed tomography at the time of PAH diagnosis.*** The chest CT shows severe dilation of the main and branch pulmonary arteries (A) and the right ventricle (B), but normal lung parenchyma (B), no thrombi, and no radiological signs for pulmonary venous occlusive disease (PVOD).

Supplementary Figure 5. Pulmonary angiogram: main and left pulmonary artery (MPA, LPA)

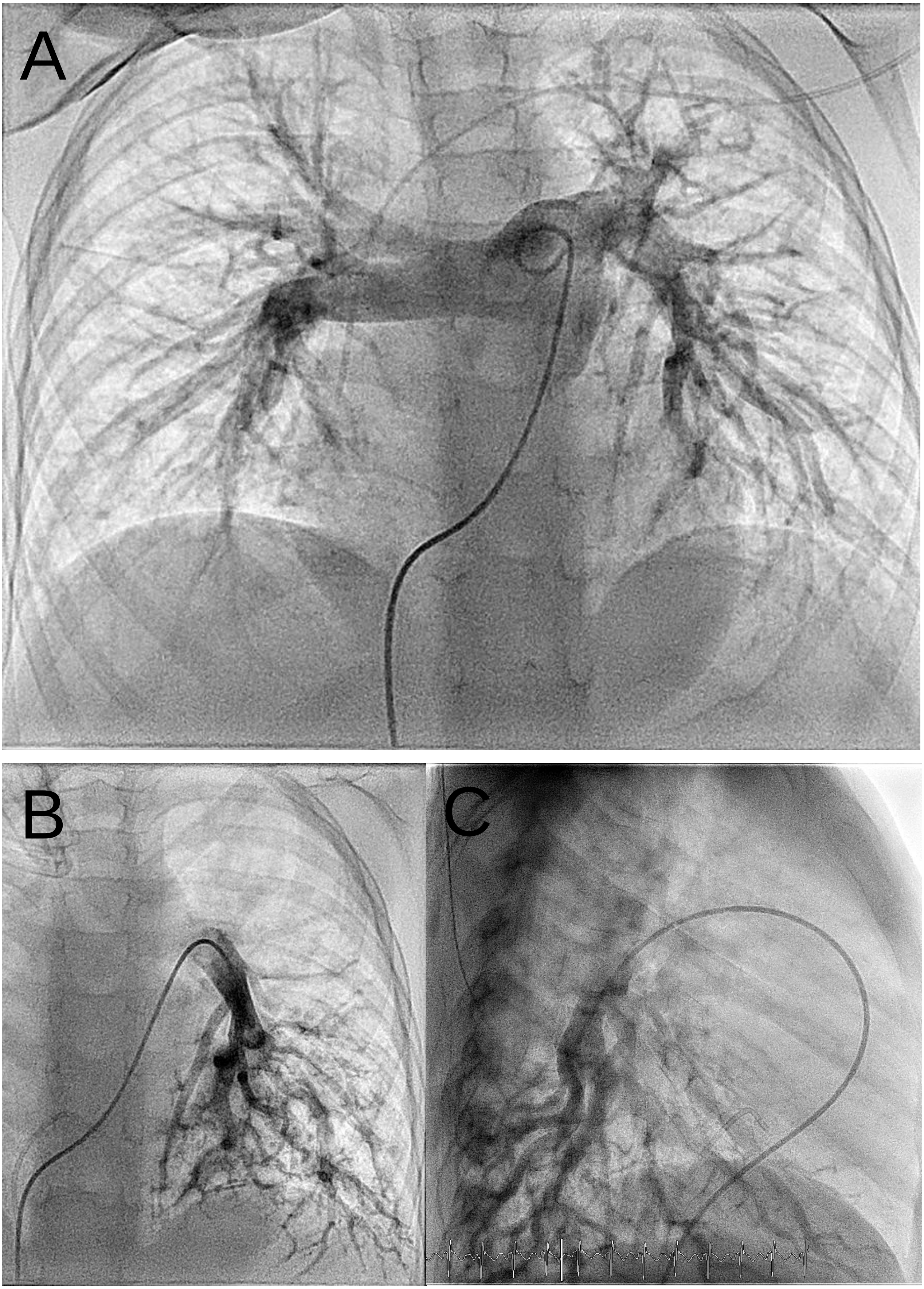

(legend on next page)

Supplementary Figure 5. Pulmonary angiograms: main and left pulmonary artery (MPA, LPA)
Contrast was injected via a pigtail catheter (4F, Terumo) placed in the main pulmonary artery (MPA; **A**) and left pulmonary artery (LPA; **B, C**). The main, right and left pulmonary arteries are dilated.
There is no prominent narrowing at the lobar and segmental branches, and no obvious pruning of the subsegmental branches.
Particularly in the selective angiograms of the branch pulmonary arteries (here LPA: **B**, posterior-anterior; **C**, lateral), the peripheral pulmonary vascular pattern appears grossly abnormal: very prominent tortuosity of the peripheral pulmonary arteries is shown. The prominent haziness of the contrast dye in the peripheral pulmonary circulation may represent diffuse (pre)capillary telangiectasia and/or very small arterio-venous malformations throughout. No obvious large pulmonary arterio-venous malformations (AVMs) are visualized (SpO_2 > 95%). See complete selective pulmonary angiograms in Supplementary movies 2A, 2B.

Supplementary Figure 6. Catheter positions during HUCMSC-CM infusions

A. Right Pulmonary Artery (RPA) **B. Left Pulmonary Artery (LPA)**

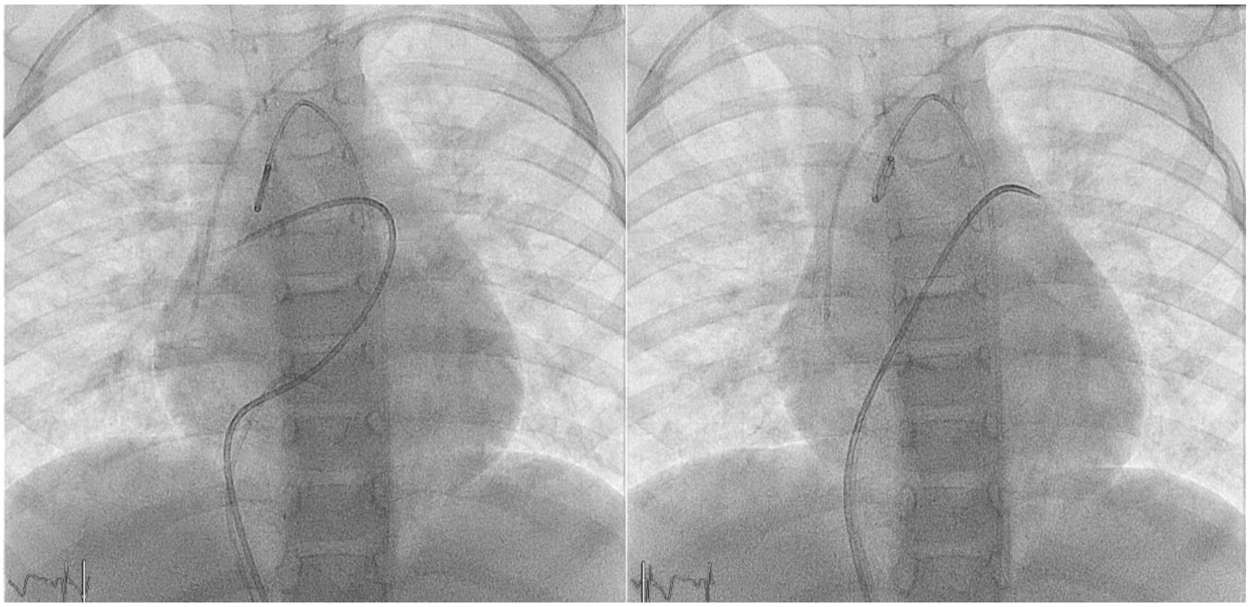

Supplementary Figure 6. *Catheter positions during HUCMSC-CM infusions.* A 5F Berman wedge catheter was placed in the right pulmonary artery (**A**) and also in the left pulmonary artery (**B**), via a right femoral venous sheath. At time 0 and time 1 (2 months later), HUCMSC-CM over were infused into the right pulmonary artery (100mL/min.) and into the left pulmonary artery (100mL/30min.). A 4F pigtail catheter (Terumo) was placed via a right femoral venous sheath in the ascending aorta (AAO) for hemodynamic assessment and monitoring. A third femoral sheath (4F, Terumo) was placed in the left femoral vein for hemodynamic assessment (SVC, RA, IVC; 4F Cordis Cobra catheter not shown). For the serial central intravenous HUCMSC-CM infusions (following the intrapulmonary arterial HUCMSC-CM infusions in the cardiac catheterization laboratory), a 4.5F catheter (Vygon Multicath 3, 3-lumen) is placed under ultrasound guidance in the left basilic vein (forearm), with the tip ending at the junction of the superior vena cava (SVC) and the right atrium (RA). The catheter positions with labeling and the time course of HUCMSC-CM infusions are shown in Figure 1A of the main manuscript.

Supplementary Figure 7. Echocardiography indicates improved RV-LV-interaction 6 months after start of HUCMSC-CM therapy

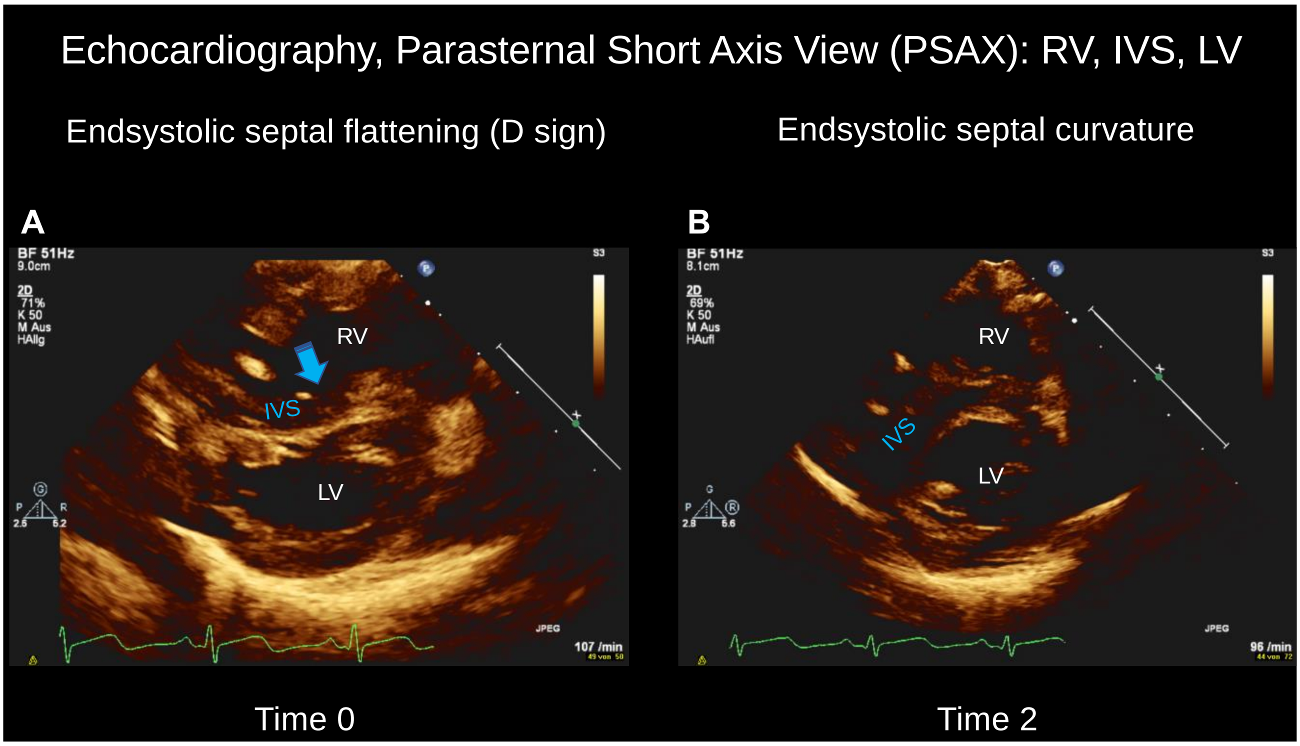

Supplementary Figure 7. ***Echocardiography indicates improved RV-LV interaction 6 months after the start of HUCMSC-CM therapy.*** Transthoracic echocardiographic B mode still frames in the parasternal short axis view (PSAX) at baseline (**A**, Time 0) and 6 months after the start of HUCMSC-CM therapy (**B**, Time 2). The images are taken at end-systole (T wave), and show a hypertrophied right ventricle (RV) and a non-hypertrophied left ventricle (LV). Assessment of the end-systolic curvature of the interventricular septum (IVS) reveals in **A** (baseline, time 0) complete flattening of the IVS (D-sign), indicating systemic RV and pulmonary arterial pressure (PAP), consistent with a mPAP/mSAP ratio of 0.94 that was determined under analgosedation on the subsequent day in the cardiac catheterization laboratory. Six months after the start of HUCMSC-CM therapy (**B**, time 2), the IVS curvature in end-systole is not flat anymore but rather convex to the RV, indicating sub-systemic RV and pulmonary arterial pressure, consistent with a mPAP/mSAP ratio of 0.81 determined under analgosedation on the subsequent day in the cardiac catheterization laboratory. See also in the main manuscript Figure 1E for mPAP/mSAP ratios and Figure 1H for RV/LV end-systolic ratios over time.

Supplementary Figure 8. Single-cell RNA sequencing of HUCMSC reveals four MSC clusters

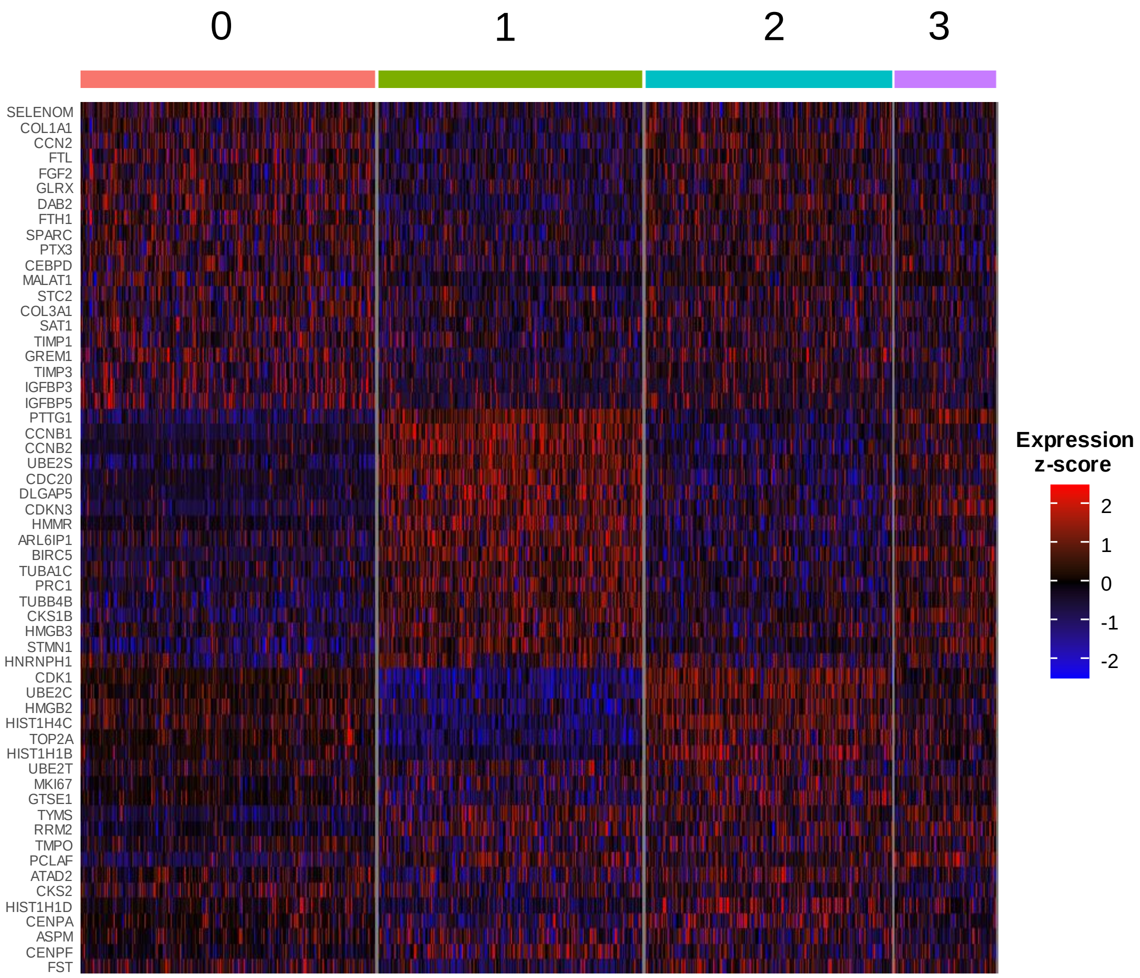

Supplementary Figure 8. Single-cell RNA sequencing of HUCMSCs (whose conditioned media was used for treatment of the PAH patient) reveals four MSC clusters. The heatmap of the marker gene expression separates the cells into functionally different subpopulations. The subpopulations were identified based on the expression profiles of all sequenced cells. The marker genes represent the upregulated genes of the three cell clusters (0 - 2). Cluster 3 had no upregulated genes.

Supplementary Figure 9. Prostaglandin E_2 (PGE_2) synthesis enzymes are ubiquitously expressed in the majority of all four MSC cell clusters as opposed to the enzymes that synthesize PGI_2 and PGD_2, in the HUCMSC-CM treated pediatric PAH patient

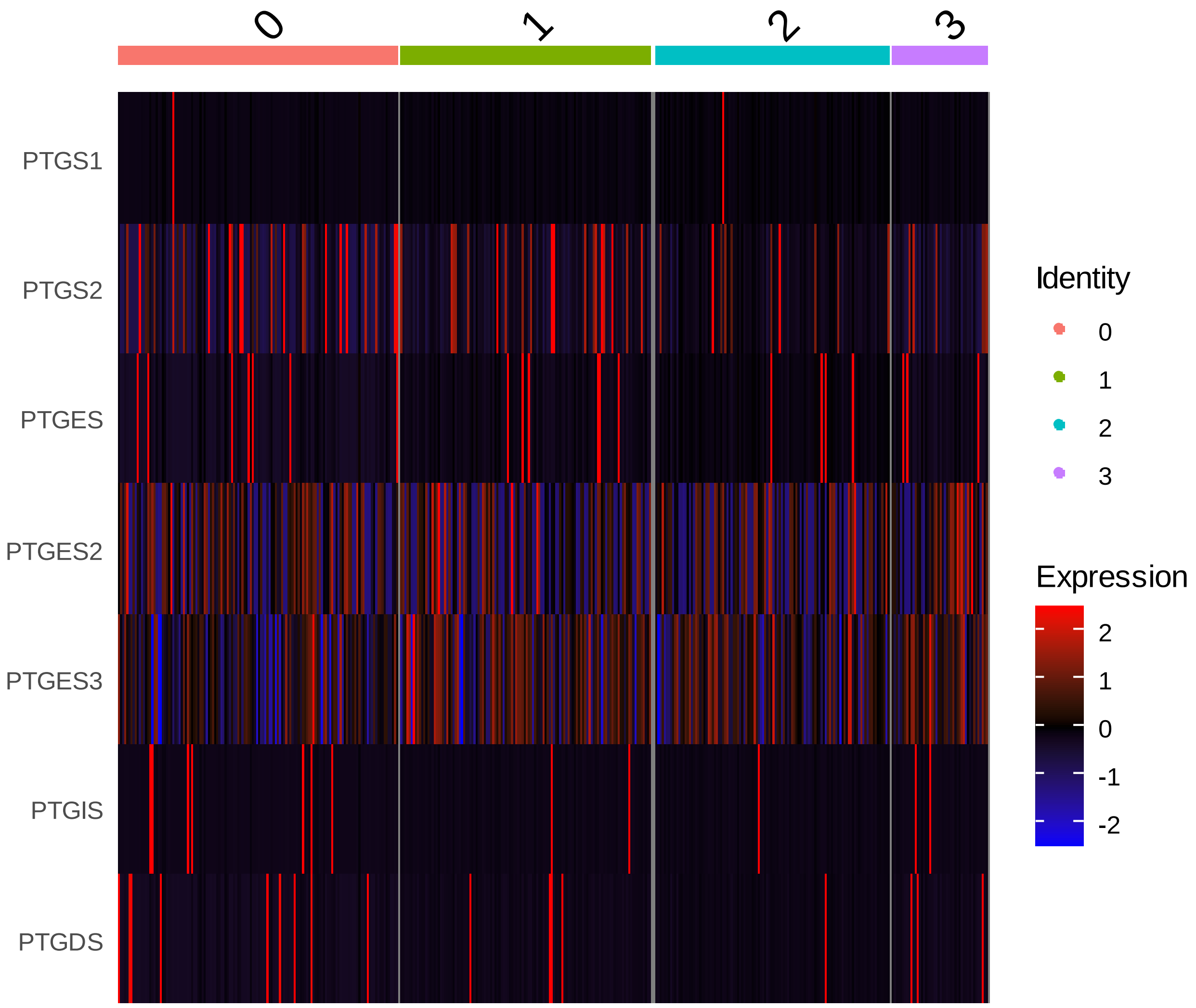

Supplementary Figure 9. Prostaglandin E_2 (PGE_2) synthesis enzymes are ubiquitously expressed in the majority of the MSC cells as opposed to the enzymes that synthesize PGI_2 and PGD_2, in the HUCMSC-CM treated PAH patient. Each column in the heatmap represents a single cell. The expression levels are shown as log2(z-score) for each individual gene. Genes with mostly black background have very small expression range with the mean expression level for the entire cell population close to absence of expression. Prostaglandin-endoperoxide synthase 1 (PTGS1) and 2 (PTGS2) encode cyclooxygenase-1 (COX1) and cyclooxygenase-2 (COX2), the enzymes that convert free arachidonic acid into prostaglandin PGH_2. Three prostaglandin E synthases, i.e. PTGES, PTGES2, and PTGES3, are enzymes involved in synthesis of prostaglandin PGE_2. Prostaglandin I synthase (PTGIS) and prostaglandin D synthase (PTGDS) are involved in synthesis of prostaglandins PGI_2 and PGD_2, respectively.

SUPPLEMENTARY MOVIES

Supplementary Movies 1 A, B. Pulmonary artery angiograms in the left pulmonary artery. (**A**: posterior-anterior, **B**: lateral).
Contrast was injected via a pigtail catheter (4F, Terumo) placed **left lower lobe pulmonary artery**. There is no prominent narrowing at the lobar and segmental branches, and no obvious pruning of the subsegmental branches.
The peripheral pulmonary vascular pattern appears grossly abnormal: very prominent tortuosity of the peripheral pulmonary arteries is shown. The prominent haziness of the contrast dye in the peripheral pulmonary circulation may represent diffuse (pre)capillary telangiectasia and/or very small arterio-venous malformations throughout. No obvious large pulmonary arterio-venous malformations (AVMs) are visualized (SpO_2 > 95%). See complete selective pulmonary angiograms in Supplementary movies 2A, 2B.
On pulmonary venous recirculation, contrast returns normally via the regularly sized left lower lobe pulmonary vein to the left atrium. There is no obvious flow of contrast from the left atrium to the right atrium (no significant atrial shunt). See also Supplementary Figure 5 for main and left pulmonary artery angiogram, and Supplementary Figure 6 for catheter positions during HUCMSC-CM infusions.

Supplementary Movies 2 A, B. Transthoracic echocardiography (B mode): Parasternal short axis view (PSAX) at baseline (Time 0) and 6 months after the start of HUCMSC-CM therapy (Time 0).
Assessment of the end-systolic curvature of the interventricular septum (IVS) shows in **A** (baseline, time 0) complete flattening of the IVS (D-sign), indicating systemic right ventricular pressure, consistent with a mPAP/mSAP ratio of 0.94 determined on the subsequent day in the cardiac catheterization laboratory.
Six months after the start of HUCMSC-CM therapy (**B**, time 2), the IVS curvature in end-systole is not flat but rather convex to the RV, indicating sub-systemic RV and PA pressure, consistent with a mPAP/mSAP ratio of 0.81 determined on the subsequent day in the cardiac catheterization laboratory. See also in the main manuscript Figure 1E for mPAP/mSAP ratios and Figure 1H for RV/LV end-systolic ratios over time. The according still frames, taken in end-systole, are shown in Supplementary Figure 5.

SUPPLEMENTARY TABLES

Supplementary Table 1. Characteristics of PAH patient at baseline (Cath #1) and follow up (Caths #2-3) – extended data set

	**Baseline**<br><br>**Cath #1**<br>12/2019	**s/p 1 dose**<br>**MSC-CM**<br>**Cath #2**<br>02/2020	**s/p 5 doses**<br>**MSC-CM**<br>**Cath #3**<br>06/2020	**Percent change (Cath #3 vs. #1)**
**Demographics**				
Age (years)	3.9	4.1	4.4	
**Height (m)**	**0.96 (5th Perc.)**	**1.06 (65th Perc.)**	**1.06 (45th Perc.)**	**+10%**
**Weight (kg)**	**12.6 (2nd Perc.)**	**14.5 (15thPerc.)**	**15.1 (15thPerc.)**	**+20%**
BSA (m^2)	0.58	0.65	0.67	+16%
**Clinical Diagnosis**				
PH Group	HPAH, ACVRL 1 mutation (PH group 1.2)	HPAH, ACVRL 1 mutation (PH group 1.2)	HPAH, ACVRL 1 mutation (PH group 1.2)	
Co-morbidities	HHT, avWS type 2, thrombocytopenia	HHT, avWS type 2	HHT, avWS type 2	
**Functional Status**				
WHO Functional Class	3	2	1	-2
**6-min. walk distance (m)**	**370**	**450**	**485**	**+31%**
**Risk Stratification (EPPVDN)**				
Risk Category (EPPVDN)	Intermediate Risk	Intermediate Risk	Intermediate Risk	
Higher Risk Score	3/21 (**0.14**)	1/21 (**0.05**)	1/21 (**0.05**)	**-64%**
Lower Risk Score	13/20 (**0.65**)	16/20 (**0.80**)	16/20 (**0.80**)	**+23%**
**Hemodynamics**				
Cardiac catheterization				
Date	December, 2019	February, 2020	June, 2020	
MSC-CM doses received	0	1	5	
Months since 1st MSC-CM	0	2	5	
Months since 2nd MSC-CM	N/A	0	3	
mRAP (mm Hg)	5	3	4	
RVEDP (mm Hg)	11	8	9	
sPAP (mm Hg)	85	72	67	
**mPAP (mm Hg)**	**59**	**52**	**48**	**-19%**
**dPAP (mm Hg)**	**38**	**31**	**29**	**-24%**
sSAP/mSAP/dSAP (mmHg)	82/63/44	81/59/40	78/59/43	
**mPAP/mSAP**	**0.94**	**0.88**	**0.81**	**-14%**
**dPAP/dSAP**	**0.86**	**0.78**	**0.67**	**-22%**
PAWP (mm Hg)	7	6	6	
LVEDP (mm Hg)	14	-	-	
**mTPG (mm Hg)**	**52**	**46**	**42**	**-19%**
**dTPG (mm Hg)**	**31**	**25**	**23**	**-26%**
PVRi (WU·m^2)	10.6	6.7	10.1	
SVRi (WU·m^2)	11.4	8.8	12.6	
**PVR/SVR**	**0.93**	**0.76**	**0.80**	-14%
Qpi (L/min/m^2)	4.9	6.91	4.16	
Qsi (L/min/m^2)	4.9	6.37	4.37	
Qp/Qs	1.0	1.08	0.95	

Supplementary Table 1 (continued)

Echocardiography				
Date	December, 2019	February, 2020	June, 2020	
Heart Rate (bpm)	94	97	97	
RVAWD (cm), PSAX	0.4	0.5	0.4	
RVEDD (cm), PSAX	1.3	1.4	1.4	
**RV/LV end-systolic ratio, PSAX**	**1.60**	**0.94**	**0.86**	**-46%**
LV eccentricity index, PSAX	**1.39**	**1.42**	**1.23**	-12%
**S/D ratio (TRV jet)**	**1.85**	**1.31**	**1.23**	**-34%**
**TAPSE (cm), apical**	**1.64 (z -0.77)**	**2.1 (z +2.15)**	**2.2 (z +2.92)**	**+34%**
PAAT (ms), PSAX	78 (z -2.31)	100 (z -0.77)	94 (z -1.35)	+21%
LVEF (%)	58	60	65	+12%

Cardiac MRI				
Date	-	February, 2020	June, 2020	
Heart rate (bpm)		97	117	
RVEDV (ml/m²)		72	79	+10%
RVESV (ml/m²)		33	29	-12%
**RV stroke volume (ml m²)**		**39**	**50**	**+28%**
**RV output, Qpi (l/min/m²)**		**3.78**	**5.85**	**+55%**
LVEDV (ml/ m²)		83	83	
LVESV (ml/ m²)		28	28	
LV stroke volume (ml/ m²)		66	67	
LV output, Qsi (l/min/ m²)		6.4	7.84	
**RVEF (%)**		**54**	**63**	**+17%**
LVEF (%)		66	67	
RV mass (g/ m²)		33	41	

Supplementary Table 1. Demographics and hemodynamic data of HPAH patient before, during and after infusion of human umbilical cord mesenchymal stem cell conditioned media (HUCMSC-CM) via an intrapulmonary arterial catheter (total of 2 doses, 200mL each) and a central venous catheter (total of 3 doses, 200mL each).

The first cardiac catheterization (*CATH #0; at diagnosis, treatment naïve*) was conducted in January 2019 at an external tertiary center. Invasive hemodynamic measurements demonstrated severe PAH with suprasystemic PA pressure (PAP 119/57/85 mmHg, AAO 94/43/63 mmHg, mPAP/mSAP = 1.35) and severely elevated pulmonary vascular resistance (PVRi 21 WU*m²; PVR/SVR ratio 1.2; cardiac index (Qsi) 3.6L/min/m²). Accordingly, at diagnosis, patient reached "high risk" stratification, with an EPPVDN higher risk score of 12/15 (0.8; non-invasive) and 14/21 (0.67; combined non-invasive + invasive hemodynamics), and lower risk scores of 0/14 (0) and 4/20 (0.2; combined non-invasive + invasive hemodynamics

Abbreviations: BSA, body surface area; D, diastolic; dPAP, diastolic pulmonary arterial pressure; dTPG, diastolic transpulmonary pressure gradient; HHT, hereditary hemorrhagic telangiectasia; HPAH, hereditary pulmonary hypertension; IPAH, idiopathic pulmonary hypertension; LV, left ventricle; LVEDP, left ventricular end-diastolic pressure; LVEDV, left ventricular end-diastolic volume; LVEF, left ventricular ejection fraction; mPAP, mean pulmonary arterial pressure; mRAP, mean right atrial pressure; mSAP, mean systemic arterial pressure; mTPG, mean transpulmonary pressure gradient; PAAT, pulmonary artery acceleration time; PAWP, pulmonary arterial wedge pressure; PAH, pulmonary arterial hypertension; PH, pulmonary hypertension; PSAX, parasternal short axis; PVR, pulmonary vascular resistance; PVRi, pulmonary vascular resistance index; Qpi, pulmonary blood flow index; Qsi, systemic blood flow index (= cardiac index); RV, right ventricle; RVAWD, right ventricular anterior wall diameter; RVEDD, right ventricular end-diastolic diameter; RVEDP, right ventricular end-diastolic pressure; RVEDV, right ventricular end-diastolic volume; RVEF, right ventricular ejection fraction; S, systolic; sPAP, systolic pulmonary arterial pressure; sSAP, systolic systemic arterial pressure; SVR, systemic vascular resistance; TAPSE, tricuspid annular plane systolic excursion; TRV, tricuspid regurgitation velocity; avWS, acquired von Willebrand syndrome type 2

Supplementary Table 2. Functional annotation of the human umbilical cord MSC subpopulations

Cluster	MarkerSet0	MarkerSet1	MarkerSet2	Annotation
0	++	-	+/-	Regenerative cells
1	-	++	--	Proliferative cells
2	+	-	++	Proliferative cells with regeneration potential
3	-	+	+/-	Proliferative cells
	Regeneration	Mitosis	Metabolic processes	
	ECM	Cell cycle	Mitosis	
	Collagen		DNA repair	
	Angiogenesis			

Supplementary Table 2. Functional annotation of the MSC subpopulations in the cells whose conditioned media was used for treatment of the reported PAH patient. Only three sets of marker genes (upregulated differentially expressed genes (DEGs)) are used, because Cluster 3 had only downregulated DEGs. Pluses and minuses represent strength of the gene expression for the overall set of genes within a cluster. The annotation categories are based on GO biological process and/or BioPlanet pathway overrepresentation associated with the corresponding marker gene set.
