# Supplementary material for: Human umbilical cord mesenchymal stem cell-derived treatment of severe pulmonary arterial hypertension
Source: Nat Cardiovasc Res. 2022 Jun 9;1(6):568–76. doi: 10.1038/s44161-022-00083-z (PMC11358026; doi:10.1038/s44161-022-00083-z)
Supplement: Supplementary file 2 — Reporting Summary [file 44161_2022_83_MOESM2_ESM.pdf]

nature portfolio

Corresponding author(s): Georg Hansmann

Last updated by author(s): Jan 11, 2022

Reporting Summary

Nature Portfolio wishes to improve the reproducibility of the work that we publish. This form provides structure for consistency and transparency in reporting. For further information on Nature Portfolio policies, see our Editorial Policies and the Editorial Policy Checklist.

Statistics

For all statistical analyses, confirm that the following items are present in the figure legend, table legend, main text, or Methods section.

n/a	Confirmed	
☐	☒	The exact sample size (n) for each experimental group/condition, given as a discrete number and unit of measurement
☐	☒	A statement on whether measurements were taken from distinct samples or whether the same sample was measured repeatedly
☐	☒	The statistical test(s) used AND whether they are one- or two-sided<br>*Only common tests should be described solely by name; describe more complex techniques in the Methods section.*
☒	☐	A description of all covariates tested
☐	☒	A description of any assumptions or corrections, such as tests of normality and adjustment for multiple comparisons
☐	☒	A full description of the statistical parameters including central tendency (e.g. means) or other basic estimates (e.g. regression coefficient) AND variation (e.g. standard deviation) or associated estimates of uncertainty (e.g. confidence intervals)
☐	☒	For null hypothesis testing, the test statistic (e.g. F, t, r) with confidence intervals, effect sizes, degrees of freedom and P value noted<br>*Give P values as exact values whenever suitable.*
☒	☐	For Bayesian analysis, information on the choice of priors and Markov chain Monte Carlo settings
☒	☐	For hierarchical and complex designs, identification of the appropriate level for tests and full reporting of outcomes
☒	☐	Estimates of effect sizes (e.g. Cohen's d, Pearson's r), indicating how they were calculated

Our web collection on statistics for biologists contains articles on many of the points above.

Software and code

Policy information about availability of computer code

Data collection	scRNA-seq data:The proprietary 10x Genomics CellRanger pipeline (v4.0.0) was used with default parameters except for the setting of expected cells (--expect-cells 1500).<br>Proteomics data: DIA-NN version 1.8<br>LC-MS data: Detection was achieved on a Qtrap 6500 (Sciex Nieuwerkerk a/d IJssel, The Netherlands) equipped with a ESI source.
Data analysis	scRNA-seq data: CellRanger was used to align read data to the human reference genome provided by 10X Genomics (refdata-gex-GRCh38-2020-A) using the STAR aligner (v. 2.7.8a). Further analysis was performed using the Seurat R package (v. 4.0.2).<br>Proteomics data were analyzed using the DEP R package (v. 1.12.0).<br>Lipidomics (LC-MS) results were analyzed using GraphPad Prism v. 7.<br>GO term and pathway overrepresentation analysis were performed using the online tool Enrichr (https://maayanlab.cloud/Enrichr/).

For manuscripts utilizing custom algorithms or software that are central to the research but not yet described in published literature, software must be made available to editors and reviewers. We strongly encourage code deposition in a community repository (e.g. GitHub). See the Nature Portfolio guidelines for submitting code & software for further information.

Data

Policy information about availability of data

All manuscripts must include a data availability statement. This statement should provide the following information, where applicable:

- Accession codes, unique identifiers, or web links for publicly available datasets
- A description of any restrictions on data availability
- For clinical datasets or third party data, please ensure that the statement adheres to our policy

The scRNA-seq data are accessible via NCBI Gene Expression Omnibus (accession ID: GSE199071). We have deposited the raw data for proteomics experiments to PRIDE (EMBL), which is a part of ProteomeXchange (accession ID: PXD032234). The LC-MS prostaglandin data are in the Supplementary data set (Tables SD12 and SD13).

Field-specific reporting

Please select the one below that is the best fit for your research. If you are not sure, read the appropriate sections before making your selection.

☒ Life sciences ☐ Behavioural & social sciences ☐ Ecological, evolutionary & environmental sciences

For a reference copy of the document with all sections, see nature.com/documents/nr-reporting-summary-flat.pdf

Life sciences study design

All studies must disclose on these points even when the disclosure is negative.

Sample size	Clinical case and treatment is reported (n=1, Fig 1 and 2). For multi-omic analysis the sample sizes were based on availability of the tissue.<br>Number of cords for scRNA-seq analysis: HUCMSC n=3, HUVEC n=2 (Fig 3A).<br>Number of cords for proteomics and prostaglandin analyses: HUCMSC n=5, HUVEC n=4 (Fig 3C,D).
Data exclusions	No data were excluded
Replication	N/A to clinical case. Applicable to multiple cord analysis in Fig 3
Randomization	N/A to clinical case, not a randomized clinical study
Blinding	N/A not appplicable to clinical case. Fig 3 shows unbiased multi-omic assays, which were run blinded to group assignment (Fig 3).

Reporting for specific materials, systems and methods

We require information from authors about some types of materials, experimental systems and methods used in many studies. Here, indicate whether each material, system or method listed is relevant to your study. If you are not sure if a list item applies to your research, read the appropriate section before selecting a response.

Materials & experimental systems

n/a	Involved in the study	
☐	☒	Antibodies
☐	☒	Eukaryotic cell lines
☒	☐	Palaeontology and archaeology
☒	☐	Animals and other organisms
☐	☒	Human research participants
☒	☐	Clinical data
☒	☐	Dual use research of concern

Methods

n/a	Involved in the study	
☒	☐	ChIP-seq
☒	☐	Flow cytometry
☒	☐	MRI-based neuroimaging

Antibodies

Antibodies used	NEDD9 Detector Antibody (Aviva Systems Biology, San Diego, CA, OKEH02459, Lot KE0777); Plasma ICAM-1 (sample dilution 1:1000), SAA (sample dilution 1:1000), IFN-γ (sample dilution 1:2) concentrations were measured by applying Meso Scale Discovery's Multi-Array technology (Vascular Injury (Catalog No K15198D) and Proinflammatory Panels (Catalon No K15049D).
Validation	Commercial product assays already validated (per manufacturer's instructions)

Eukaryotic cell lines

Policy information about cell lines

Cell line source(s)	primary human mesenchymal stroma/stem-like cells (MSC) derived from umbilical cord
Authentication	N/A
Mycoplasma contamination	no mycoplasma contamination tested
Commonly misidentified lines (See ICLAC register)	N/A

Human research participants

Policy information about studies involving human research participants

Population characteristics	Sample size n=1. At diagnosis, the 3-year-old girl was in critical condition, status post two syncopal, “afebrile seizure episodes”, in WHO functional class 4, with a 6 minute-walking-distance of only 270 meters (SpO2>95%), and moderate thrombocytopenia at 80·103/mcL. She had a 10 months history of fatigue, repetitive nose bleeding (epistaxis), and mucocutaneous telangiectases at the lips, chest and lower extremities.
Recruitment	Single case. The caregivers (parents) of the patient gave written informed consent (compassionate use, therapy, publication).
Ethics oversight	The use of primary human MSCs following explant culture from umbilical cord tissue (#443) and RNA/protein expression studies (#2200) have been approved by the Ethics Committee of Hannover Medical School.

Note that full information on the approval of the study protocol must also be provided in the manuscript.
